# DNase I hypersensitivity analysis of the mouse brain and retina identifies region-specific regulatory elements

**DOI:** 10.1186/1756-8935-8-8

**Published:** 2015-02-28

**Authors:** Matthew S Wilken, Joseph A Brzezinski, Anna La Torre, Kyle Siebenthall, Robert Thurman, Peter Sabo, Richard S Sandstrom, Jeff Vierstra, Theresa K Canfield, R Scott Hansen, Michael A Bender, John Stamatoyannopoulos, Thomas A Reh

**Affiliations:** Department of Biological Structure, University of Washington, 1959 NE Pacific Street, Box 357420, Seattle, WA 98195 USA; Department of Genome Sciences, University of Washington, Foege Building S-250, 3720 15th Ave NE, Box 355065, Seattle, WA 98195 USA; Molecular and Cellular Biology Program, University of Washington, MCB Program Office, T-466 Health Sciences Building, Box 357275, Seattle, WA 98195 USA; Department of Pediatrics, University of Washington, 1959 NE Pacific St, Health Sciences Building, Seattle, WA Box 356320, 98195 USA; Clinical Research Division, Fred Hutchinson Cancer Research Center, 1100 Fairview Avenue North, Seattle, WA 98109 USA; Department of Ophthalmology, University of Colorado School of Medicine, 1675 Aurora Court, Aurora, CO 80045 USA

**Keywords:** Central nervous system, Retina, DNase-seq, Cis-regulation

## Abstract

**Background:**

The brain, spinal cord, and neural retina comprise the central nervous system (CNS) of vertebrates. Understanding the regulatory mechanisms that underlie the enormous cell-type diversity of the CNS is a significant challenge. Whole-genome mapping of DNase I-hypersensitive sites (DHSs) has been used to identify cis-regulatory elements in many tissues. We have applied this approach to the mouse CNS, including developing and mature neural retina, whole brain, and two well-characterized brain regions, the cerebellum and the cerebral cortex.

**Results:**

For the various regions and developmental stages of the CNS that we analyzed, there were approximately the same number of DHSs; however, there were many DHSs unique to each CNS region and developmental stage. Many of the DHSs are likely to mark enhancers that are specific to the specific CNS region and developmental stage. We validated the DNase I mapping approach for identification of CNS enhancers using the existing VISTA Browser database and with *in vivo* and *in vitro* electroporation of the retina. Analysis of transcription factor consensus sites within the DHSs shows distinct region-specific profiles of transcriptional regulators particular to each region. Clustering developmentally dynamic DHSs in the retina revealed enrichment of developmental stage-specific transcriptional regulators. Additionally, we found reporter gene activity in the retina driven from several previously uncharacterized regulatory elements surrounding the neurodevelopmental gene *Otx2*. Identification of DHSs shared between mouse and human showed region-specific differences in the evolution of cis-regulatory elements.

**Conclusions:**

Overall, our results demonstrate the potential of genome-wide DNase I mapping to cis-regulatory questions regarding the regional diversity within the CNS. These data represent an extensive catalogue of potential cis-regulatory elements within the CNS that display region and temporal specificity, as well as a set of DHSs common to CNS tissues. Further examination of evolutionary conservation of DHSs between CNS regions and different species may reveal important cis-regulatory elements in the evolution of the mammalian CNS.

**Electronic supplementary material:**

The online version of this article (doi:10.1186/1756-8935-8-8) contains supplementary material, which is available to authorized users.

## Background

The human central nervous system (CNS; brain, spinal cord, and neural retina of the eye) contains billions of neurons, with hundreds of distinct types. Studies of neuronal morphology, neurotransmitter response, and single-cell electrophysiological analyses have traditionally been used to define neuronal diversity and led to estimates of different types of neurons ranging in the thousands. Recent large-scale molecular mapping studies, however, have shown an even greater complexity than that previously appreciated [1–3]. The enormous diversity in gene expression and connectivity in the brain presents a challenge for traditional approaches to define regulatory networks and identify cis-regulatory elements active in this complex organ.

Several previous studies have used comparative genomics approaches to identify cis-regulatory modules (CRMs) in both developing and mature CNS, based on the fact that these are frequently conserved across species [4–7]. More recently, epigenetic approaches have used stereotyped patterns of histone modifications and the occupancy of DNA-binding proteins to identify various types of CRMs [8–13]. Combinations of these approaches have also been effective, particularly in identifying gene promoters. Promoters are typically found within 100 bp of the transcription start site, associate with RNA polymerase II, and frequently contain distinct sequence motifs such as the TATA box. Other types of CRMs, such as enhancers and insulators, have been somewhat more difficult to identify; however, the former are often bound by the transcriptional co-activator P300 and the latter by the zinc-finger transcription factor CTCF. In addition, characteristic histone modifications, such as H3K4me1 (enhancers) and H3K4me3 (promoters), are also good predictors of specific types of CRMs [14].

While these advances have generated large numbers of potential CRMs, there are many reasons to suspect that this list of candidates is not yet comprehensive. Although many CRMs show substantial sequence conservation among species, recent estimates suggest that nearly 40% of CRMs active in the mouse are not active in humans, despite their conserved sequence, highlighting the limitation of inferring function from comparative sequence methods alone [15, 16]. In addition, certain patterns of histone modifications and DNA-binding protein occupancy are well correlated with active enhancers and insulators; however, CRMs utilizing alternate molecular mechanisms will be missed by approaches relying solely on these patterns.

To generate a more comprehensive view of CRMs, the use of DNase I hypersensitivity mapping at the genome scale (DNase-seq) has emerged as a powerful approach [17–22]. DNase I-hypersensitive sites (DHSs) are sensitive markers of all of the main types of CRMs, and recent genome-wide mapping of DHSs in diverse human and mouse cell lines and tissues has generated fundamental insights into gene regulation and its evolution [19].

We therefore undertook a genome-scale, high-resolution mapping of accessible chromatin using DNase-seq to identify CRMs utilized *in vivo* in the developing and mature mouse brain and three specific regions, the cerebral cortex, the cerebellum, and the developing neural retina. By comparing CNS DHSs with DHSs active in other mouse cell lines and tissues, we were able to delineate a core ‘regulome’ for the CNS. We were also able to carry out an analysis of transcription factor binding motifs in CRMs active within the developing and mature retina to identify stage-specific transcriptional regulators and confirmed that a number of these potential CRMs display enhancer activity *in vitro* and *in vivo*. Overall, our results demonstrate the power of genome-wide DNase I mapping to provide answers to questions of neuronal diversity, brain evolution, and the cis-regulation that underlies these processes.

## Results

### Broad features of the regulatory landscape of mouse CNS

We carried out DNase-seq (according to ENCODE standard protocols; see Methods and [22, 23] for mature mouse whole brain, and dissected cerebral cortex, cerebellum, and neural retina (age: 8-week adult), as well as specific ages of developing brain and retina. Samples were prepared in duplicate, except where noted. We identified regions of increased DNase I-hypersensitivity called hotspots (see Methods) with a false discovery rate of less than 1% and specific 150-bp peaks within these regions. Approximately 100,000 to 250,000 DHS peaks were mapped in each sample (Additional file 1: Table S1).

**Figure 1:**
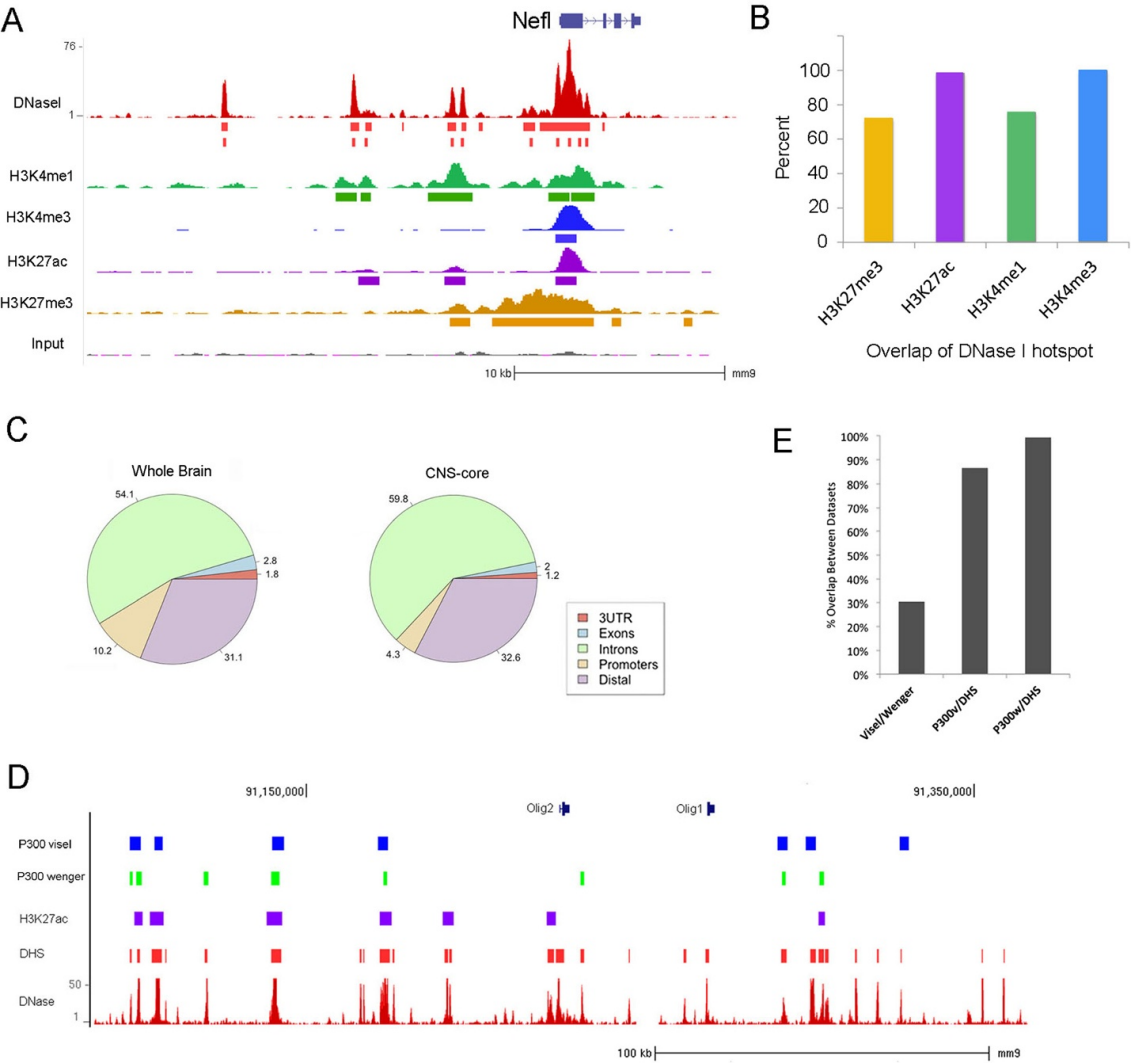
**Global analysis of the DHS landscape of mouse CNS. (A)** Comparison of DHSs to other epigenetic marks at the *Nefl* locus. DNase I cleavage patterns are shown for the E14.5 mouse brain (red) and ChIP-seq for H3K4me1 (green), H3K4me3 (blue), H3K27ac (purple), and H3K27me3 (yellow). Solid bars under the ChIP-seq signals represent peak calls. The top row of solid bars under the DNase I signal represents hotspot calls (see Methods), and the bottom row represents DHS peak calls. **(B)** The percentage of ChIP-seq peaks of different histone modifications that overlap with DHSs in E14.5 brain. DHSs from the E14.5 mouse brain to the H3K27ac ChIP-seq data at the same age shows close to 100% (99.6%) of the H3K27ac sites overlap with those identified by a DHS. **(C)** Distribution of DHSs present in adult brain and in CNS-core DHSs relative to genomic features. **(D, E)** Comparison of DNase I hypersensitivity in E14.5 mouse brain to recent P300 or H3K27ac ChIP-seq [15, 28, 29] studies of developing mouse cerebral cortex. **(D)** Correspondence between DNase I hypersensitivity, H3K27ac ChIP-seq, and two P300 ChIP-seq experiments. Peak calls for these three related studies near the *Olig1* and *Olig2* genes, along with the DNase I hotspots for E14.5 brain. P300 visel, peak calls for P300 ChIP-seq performed by [28]; P300 wenger, peak calls for P300 ChIP-seq performed by [29]; H3K27ac, peak calls for H3K27ac ChIP-seq [15]; DHS, DNase I hotspots; DNase, DNase I signal track. **(E)** Genome-wide comparisons for correspondence of the P300 ChIP-seq and DNase I hotspots showing >80% of the P300 peaks overlap with DNase I hotspots. Bar values indicate the percent of overlap between the indicated datasets.

The brain and retina DHSs overlapped with many previously identified cis-regulatory regions of the genome [14]. An example of this overlap is shown in Figure 1A for the neurofilament gene, *Nefl*, a gene expressed throughout the nervous system. In the embryonic day 14.5 (E14.5) mouse brain, DNase I-hypersensitive regions align with ChIP-seq peaks from the EMCODE project [24] for marks of promoters (H3K4me3), enhancers (H3K4me1, H3K27ac), and poised or negatively regulated regions (H3K27me3) (Figure 1A). When we compared the overlap of DHSs, pairwise, with different epigenetic modifications across the whole genome, we found that their overlap ranged from 72% (H3K27me3) to 99% (H3K27ac), depending on the particular mark (Figure 1B). The overlap of brain DHSs with genomic features is shown in Figure 1C (Additional file 2: Figure S2A for the cerebral cortex, the retina, and the cerebellum). Overall, the distribution of brain DHSs across the genome is similar to that of mouse DHSs present in other tissues [16], with the majority of DHSs in intronic regions (54%) or distal intergenic regions (31%).

To define DHSs unique to the CNS, we compared DHSs from the mature whole brain and mature retina with those from other mouse cell and tissue types (collectively including 1,323,372 distinct DHSs) [16]. This comparison identified 4,465 DHSs unique to the CNS (‘CNS-core’), thus defining a core ‘regulome’ of CRMs that drive gene expression in the CNS (Additional file 3: Table S2). The distribution of CNS-core DHSs relative to genomic features is similar to that of all brain DHSs, though with a decrease in promoter proximity (Figure 1C, right). We next asked (using GREAT [25]) whether there was specific enrichment of CNS-core DHSs near genes relevant to nervous system function. This gene ontology analysis showed highly significant enrichment near synaptic and axonal genes (cellular component), neurotransmitter regulation (biological process), and voltage-gated ion channels (molecular function; Additional file 2: Figure S2B). With respect to specific genes, CNS-core DHSs are located near many genes highly expressed in the nervous system, including those known to be involved in synapse formation and specificity (for example, *Dscam*, *Dscaml1*, complexins, contactins [26, 27]) and other neuronal processes (Additional file 2: Figure S2C).

### Characterization of CNS DHSs

The vast majority of DHSs in the CNS, CNS-core, and all CNS subregions are located either in introns or distal to gene transcription start sites (TSSs) and may be acting as remote enhancers [23]. To determine how the DNase I identification of putative cis-regulatory elements compares with other predictive epigenetic marks, we carried out a more detailed analysis of the developing brain. Several recent studies have characterized enhancers in embryonic mouse brain using either P300 ChIP or H3K27ac ChIP [15, 28, 29]. We compared the DNase I hypersensitivity data to these other chromatin signatures of enhancers for the E14.5 mouse brain, and the results are shown in Figure 1D. The peak calls for these three related studies are shown in Figure 1D near the *Olig1* and *Olig2* genes, along with the DNase I signal and hotspots for E14.5 brain. There is a good correspondence between the H3K27ac, the P300, and the DNase I hypersensitivity. However, there are also some regions where one P300 ChIP study shows a peak that corresponds with a DNase I hotspot which is not present in the other P300 study. There are other regions with DNase I hypersensitivity that are also identified in both the P300 ChIP studies, but not with the H3K27ac ChIP-seq. Thus, there appears to be good agreement with our DNase data and other well-characterized marks of enhancers, but the DNase I signal encompasses a wider range of potential regulatory elements.

Overall, comparing brain DHSs from E14.5 mouse to the H3K27ac ChIP-seq data at the same age (Figure 1B), we find that close to 100% (99.6%) of the H3K27ac sites overlap with those identified by DNase I hypersensitivity. Genome-wide comparisons for correspondence of the P300 ChIP-seq and DNase I analysis show that although the two different P300 ChIP studies identify somewhat different enhancers (Figure 1E), the regions identified by either P300 ChIP-seq study fall largely (87% to 94%) within the sites of DNase I hypersensitivity in the E14.5 brain (Figure 1E).

To further validate the effectiveness of the DNase I approach for identifying brain enhancers, we tested whether this method could identify brain enhancers that have already been tested in transgenic mice using the VISTA Enhancer Browser program [30]. The VISTA project has tested 435 elements from the mouse genome chosen for their high degree of sequence conservation across species and/or ChIP-seq evidence for putative enhancer marks. Of these, 94 show expression in embryonic brain. The H3K27ac ChIP-seq peaks successfully identified 58/94 of these elements, while a similar number (52/94) of these putative enhancers were identified by DNase I hotspots. Three examples are shown in Additional file 4: Figure S4.

We also tested whether DNase I would be more effective as a discriminator than H3K27ac ChIP-seq for predicting whether a putative enhancer will fail to be expressed in the brain. Of the 435 mouse elements tested, 178 failed to be expressed in any tissue and 63/178 of these non-expressed elements had H3K27ac peaks. Nearly all of the elements with H3K27ac peaks that failed to show expression in the transgenic embryo also had DHSs in the E14.5 brain (56/63; 89%). Thus, while DHSs provide an effective method for identifying putative enhancers, they are no better than H3K27ac ChIP-seq at discriminating those elements that are not confirmed by transgenic analysis.

### Region- and cell type-specific regulatory elements identified by DHSs

**Figure 2:**
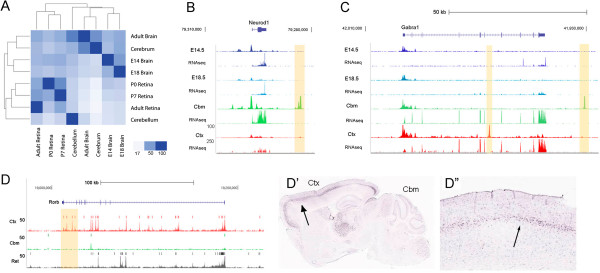
**Brain region-specific regulatory elements identified by DHSs. (A)** Heatmap of cluster analysis of the DHSs present in CNS samples clustered according to the percent of overlapping DHS peaks in each tissue, indicated by color intensity. **(B-D)** Examples of region-specific DNase I hypersensitivity landscapes with matching, previously generated RNA-seq data [24]. **(B)** DHS in the cerebellum associated with the *Neurod1* locus that is not present in the cerebral cortex (tan shading). **(C)** DHS associated with the *Gabra1* gene with examples of region-specific DHSs (tan shading). **(D)** Extensive differences in DNase I landscape (and DHS peaks, indicated as blocks over signal tracks) at the *Rorb* locus. **(D’, D”)** *Rorb* is expressed in the cerebral cortex (Ctx; arrows point to the layer of *Rorb* mRNA signal; higher magnification in **D”**) but not in the cerebellum (Cbm), shown by *in situ* hybridization (Allen Brain Atlas); there are specific DHSs associated with this gene in the cerebral cortex (tan shaded regions). E14.5, embryonic day 14.5 whole brain; E18.5, embryonic day 18.5 whole brain; Cbm, 8-week adult cerebellum; Ctx, 8-week adult cerebral cortex.

The DHS dataset is potentially useful to identify regulatory regions specific to particular CNS regions. Since many of the genes are shared between neurons, regardless of their location in the CNS, and many housekeeping genes are also likely to be shared across brain regions, comparing DHSs from different brain regions represents a potentially powerful approach to identifying neuronal subtype CRMs. To compare DHS activity between different CNS regions and developmental time points, we performed a hierarchical clustering analysis (based on the percentage of overlapping DHS peaks pairwise between each tissue) of developing and mature CNS samples (Figure 2A). As expected, the mature brain regions cluster and the developing brain samples cluster, but there are many unique DHSs between any particular CNS regions. These region-specific DHSs are present in many genes relevant to neural development and mature function. For example, there is a DHS present in the cerebral cortex associated with the *Neurod1* locus that is not present in the cerebellum (Figure 2B) even though the gene is expressed in both regions [31]. The *Gabra1* gene also shows clear examples of region-specific DHSs (Figure 2C) and again is expressed throughout the adult brain [32]. Since approximately 50% of the DHSs are potential enhancers for associated genes based on the above analysis, these region-specific DHSs provide a large list of candidate region-specific enhancers for future exploration.

We hypothesized that the transcription factors regulating neuronal and glial gene expression might differ between these region-specific DHSs and reflect the specific complement of transcriptional regulators in these different brain regions. To test for enrichment of consensus binding sites in the region-specific DHSs, we created sets of DHS peaks that are (1) present in the retina, but not in the cerebellum or cortex; (2) present in the cortex, but not in the retina or cerebellum; and (3) present in the cerebellum, but not in the retina or cortex. These sets of DHSs were analyzed with the MEME suite (DREME and CentriMo [33–35]), and we found a distinct pattern of enrichment for transcription factor motifs in the different sets (Additional file 5: Figure S5A). For example, in the retina-specific DHSs, OTX2 and CRX consensus sites were highly enriched, while in the cortex, EGR1 sites and E-box transcription factor sites predominated. Further analysis of the cortical DHSs with Centrimo of the EGR1 sites and the bHLH consensus sites show very different sets of transcription factor enrichment and gene category associations (Additional file 5: Figure S5B). CREB-related signaling pathways and glutamate receptors were associated with the EGR1 DHSs, while the bHLH binding peaks were associated with ion transport and exocytosis genes. Overall, the comparison of DHSs in different brain regions provides a powerful approach to identify new potential enhancers for neuronal and glial gene expression and the transcription factors that regulate them.

Although genome-wide DHS mapping can potentially identify all CRMs active in the CNS, because of the wide diversity of neurons, most neuronal cell types represent a relatively small fraction of the total population in any given region. Therefore, we asked whether this technique has the sensitivity to detect active regulatory elements associated with genes that are only expressed in a small percentage of cells in the CNS. We used two different approaches to address this question. First, we chose several genes that are known to be expressed in relatively sparse cell populations in the CNS and examined their promoters for the presence of DHSs. Because these genes are active in only a small number of cells, and given the strong correlation between promoter hypersensitivity and gene expression [22], this would provide a good method to evaluate the sensitivity of the DNase-seq. We queried the Allen Brain Atlas [3] for genes with laminar-specific expression, since the cortical laminae represent on average one sixth of the neurons in the cortex. We found peaks of DNase I hypersensitivity at the promoters of three laminar-specific genes, *Rorb*, *Kcnn2*, and *Etv1*, in the cerebral cortex (Figure 2D; Additional file 6: Figure S6). *Rorb* is also highly expressed in the retina [36], but not in the cerebellum, and so the region-specific DHSs are also apparent in this example. Thus, there are identifiable cortex-specific DHSs even near genes expressed in only a small percentage of the cortical neurons. However, it is important to note that these DHSs may be present in cells that do not express the gene.

**Figure 3:**
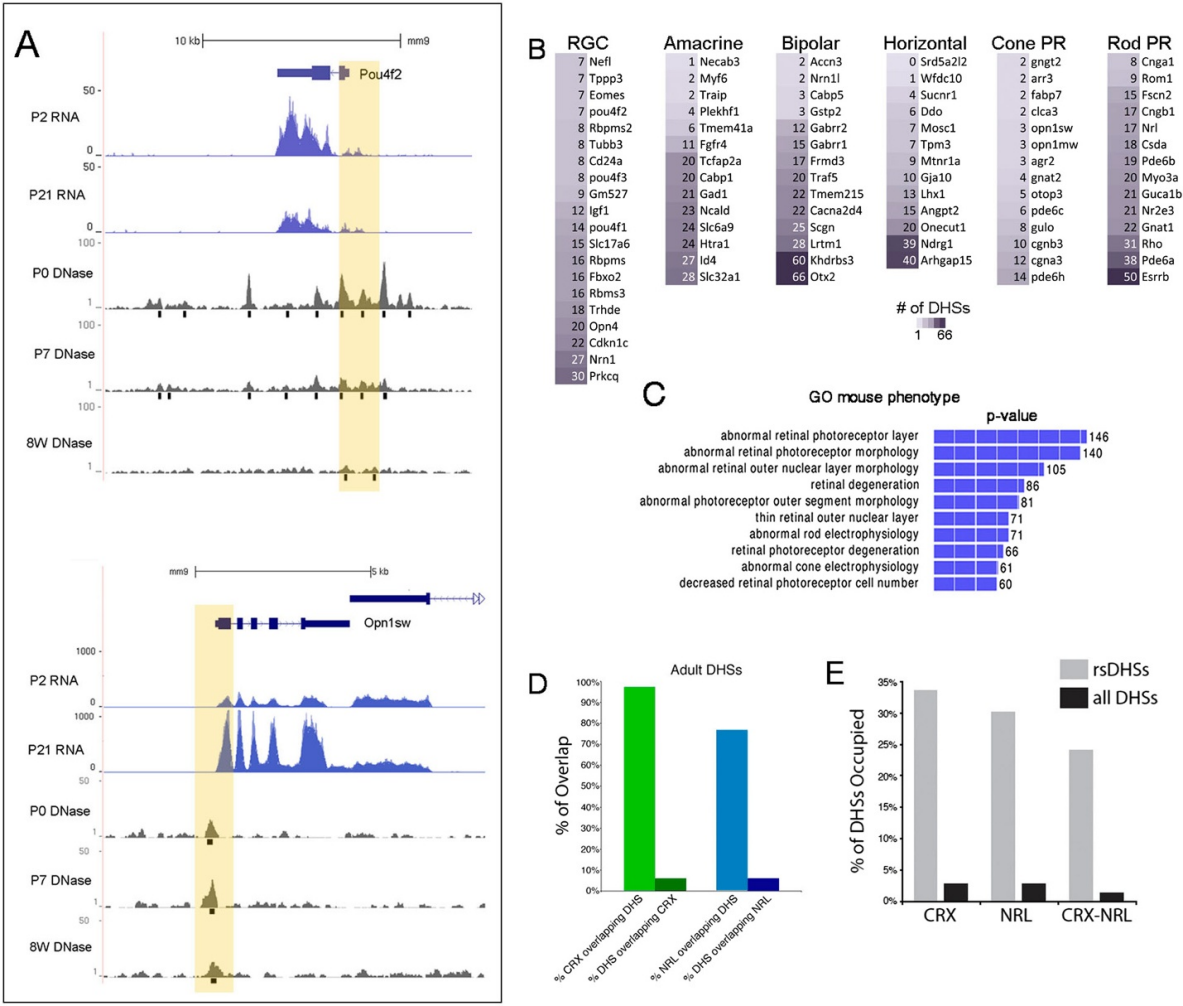
**DNase I hypersensitivity identifies (rare) cell type-specific regulatory elements. (A)** DNase I (P0, P7, and 8-week adult retina) and RNA-seq (P2 and P21 retina) [45] landscape near genes expressed exclusively in minority cell populations in the retina: ganglion cells (*Pou4f2*) and cone photoreceptors (*Opn1sw*). Black bars below DNase I signal tracks indicate DNase I peaks. Yellow boxes indicate DHS within promoter regions. **(B)** The number of DHSs near genes expressed specifically in indicated retinal cell types, sorted by column. Color intensity increases with DHS number. **(C)** Gene Ontology - Mouse Phenotype enriched terms for retinal, but not brain, DHSs as determined by GREAT analysis. Numbers indicate the number of genes associated with each term. **(D)** Overlap between ChIP-seq peaks for two key photoreceptor-specific transcription factors, CRX and NRL. Although nearly all of the ChIP-seq peaks for these TFs overlap a DHS in the retina, there are still many retinal DHSs that are associated with genes expressed in other retinal cell types. **(E)** Overlap between CRX and NRL ChIP-seq peaks (including co-binding regions) with retina-specific DHSs (rsDHSs).

To further determine whether DNase I hypersensitivity mapping can discover regulatory elements associated with expression of genes specific to particular regions of the CNS, we focused on an analysis of the neural retina, for which there has been extensive characterization of cell types and gene expression [37, 38]. Some retinal cell types, such as ganglion cells and cone photoreceptors, express genes not expressed in other regions of the CNS. Although these cell types are not highly represented in the total retinal cell population [39], we were still able to find distinct DHSs near transcription start sites and nearby enhancers of genes known to be expressed in these cells (Figure 3A); for example, *Pou4f2* is a transcription factor expressed only in a subset of retinal ganglion cells, and *Opn1sw* is a gene present exclusively in short-wavelength cone photoreceptors. The data suggest that DNase I hypersensitivity mapping can effectively identify potential cis-regulatory regions of genes expressed in specific cell types in a complex population; however, we cannot rule out that these DHSs are also present in retinal cells that do not express these genes and this will need to be tested directly in future experiments.

The results of our hierarchical clustering analysis suggested that the comparison of DHSs active in CNS regions to one another identifies region-specific regulators of genes. There were 49,383 DHSs common to the brain and retina and 51,187 DHSs in mature retina that were not in the brain; these latter DHSs were highly enriched near genes that are involved with photoreceptor and retinal phenotypes. To extend this analysis systematically, a recent gene expression characterization for specific retinal cell types purified from fluorescent reporter mice has provided ‘barcodes’ for the basic retinal cell types and many subtypes [38]. When we analyzed genes specific to each retinal cell type, we found retina-specific DHSs near these genes (Figure 3B) as determined by GREAT analysis (‘basal plus extension’ association rules [38]). We further defined a set of retina-specific DHSs (rsDHSs) by subtracting DHSs active in all other tissues and cell types in the mouse ENCODE set (38 cells/tissues) from those active in the retina (Additional file 7: Table S3). This set showed an even greater enrichment near genes known to be expressed in the retina, specifically those involved in photoreceptor function or related to retinal disease in human and mouse phenotype (Figure 3C; Additional file 8: Figure S8). Since many retina-specific DHSs would likely be associated with genes expressed in a unique retinal cell type, the photoreceptor, we compared DHSs active in the retina with the binding sites of CRX and NRL, two transcription factors essential for photoreceptor development and maintenance [40, 41]. Using previously published ChIP-seq data [42, 43], we found that 97.4% of 5,724 CRX peaks and 76.7% of 7,411 NRL peaks overlap with a DHS in the mature retina (Figure 3D). The overlaps for DHS hotspots were even greater than those for the peaks: 99.9% for CRX and 80.6% for NRL. Furthermore, a substantial fraction of retina-specific DHSs coincide with CRX or NRL binding sites or are co-bound by both factors (33%, 30%, and 24%, respectively; Figure 3E). Together, these results demonstrate that DNase-seq is a highly sensitive approach for identifying potential cis-regulatory elements that regulate CNS region-specific gene expression.

### Temporally dynamic regulatory elements

CNS development involves the processes of neurogenesis, differentiation, axon growth and pathfinding, target selection, and synaptogenesis. These processes occur primarily over the last week of fetal development and the first week of postnatal development. Overall, our hierarchical clustering analysis demonstrated that fetal brain and neonatal retina are more closely related than mature regions of the CNS. However, different brain regions have markedly different developmental stages at the same fetal age. To better analyze these processes in a sequential manner, we focused on a single CNS region (the retina) where the developmental processes are more synchronous.

**Figure 4:**
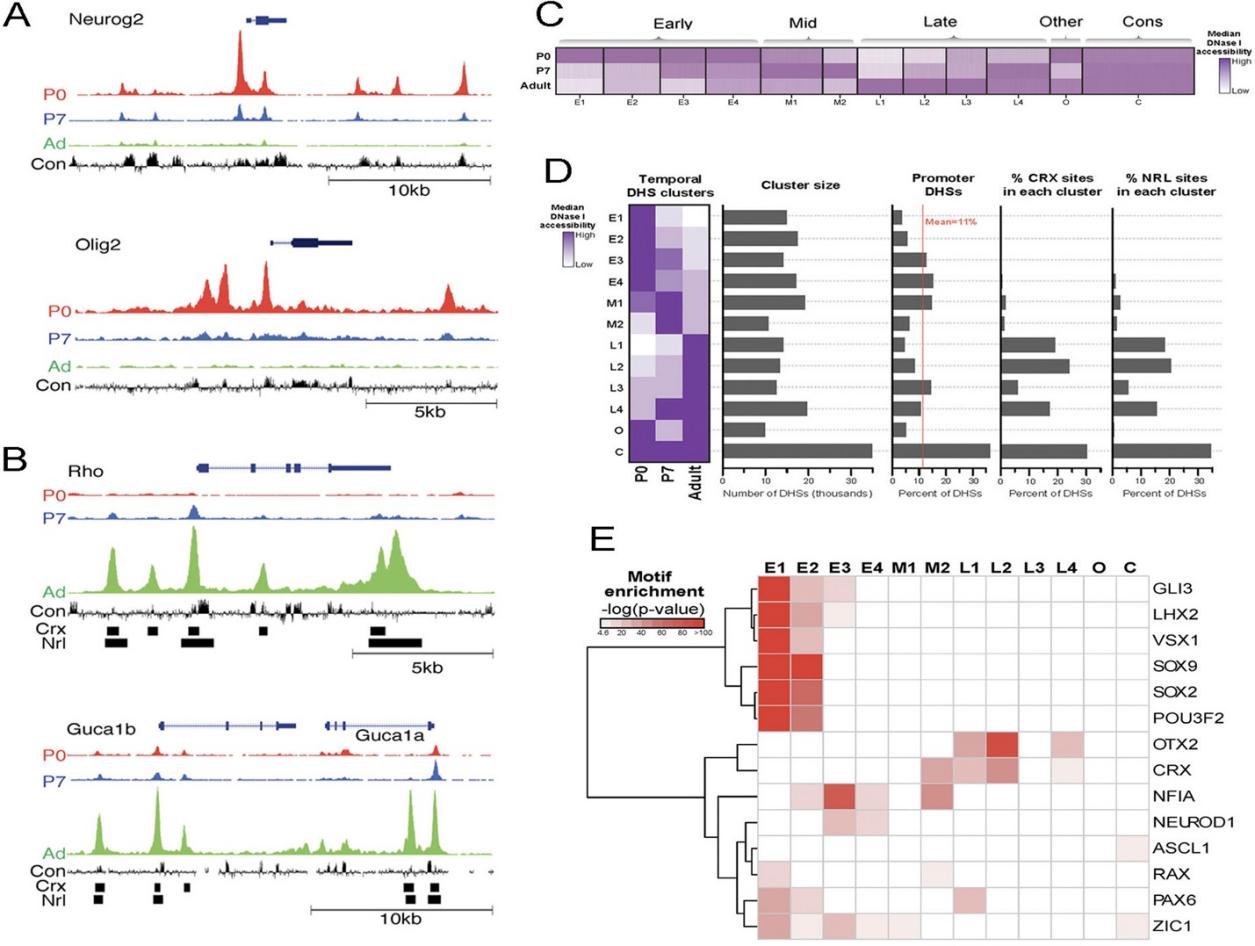
**Examination of the developmentally dynamic DNase I landscape reveals stage specific cis-regulatory elements and transcription factors.** DNase I landscape at representative gene loci near genes expressed in **(A)** developing (*Neurog2*, *Olig2*) or **(B)** mature (*Rho*, *Guca1b*) retina at P0 (red), P7 (blue), and adult (Ad; green) stages, along with mammalian sequence conservation (Con; black). ChIP-seq peak locations for CRX and NRL are indicated by black boxes [42, 43]. **(C)** Heatmap profile of accessibility at each DHS (columns) across developmental stages (rows), grouped by *k*-means clustering. Color intensity indicates the normalized DNase I accessibility according to the included scale. **(D)** Condensed heatmap of *k*-means-clustered DHSs (rows) in the retina between P0, P7, and adult stages (columns). Color intensity indicates median DNase I accessibility of DHSs in each cluster. Also shown are the number of DHSs contained in each cluster, the percentage of DHSs within each cluster that overlap a gene promoter (1 kb upstream of the transcription start site) and the percentage of total CRX or NRL ChIP-seq binding sites that overlap a DHS contained within each cluster. E, early; M, mid; L, late; O, other; C, constitutive. **(E)** Transcription factor binding motif enrichment analysis of all cluster groups shown for a selected group of transcription factors. Motif enrichment (-log10(*P* value)) indicated as color intensity for each transcription factor (rows) within each temporal cluster group (columns).

We observed clear developmentally dynamic patterns of chromatin accessibility surrounding two key developmental genes and two genes highly expressed in mature retina (Figure 4A). *Neurog2* and *Olig2*, two transcription factors expressed in retinal progenitors and necessary for neurogenesis [44], display prominent peaks of DNase I cleavage at the transcription start site and have several additional peaks in the surrounding intergenic space in P0 retina, but decrease during progression to P7 and adult retina. This corresponds to their expression patterns as demonstrated by RNA-seq (Additional file 9: Figure S9 [45]). The reverse pattern is observed for *Rho*, *Guca1a*, and *Guca1b*, genes expressed specifically in developing and mature photoreceptors [37, 46, 47]: their promoters and neighboring DHSs show substantially increasing accessibility from P0 to adult stages (Figure 4B). Again, the DHS dynamics surrounding these genes corresponds to gene expression (Additional file 9: Figure S9 [45]).

To more systematically evaluate the stage-specific dynamics of regulatory elements, we used *k*-means clustering to group all DHSs in the retina based on shared patterns of accessibility across P0, P7, and adult stages (Figure 4C). Cluster groups designated by E (early), M (mid), and L (late) contain DHSs of peak intensity at P0, P7, and adult stages, respectively. Most clusters contain approximately 10,000 to 15,000 DHSs with the exception of the constitutively accessible group, which contains 35,000 DHSs (Figure 4D). Furthermore, 5% to 15% of DHSs in each cluster are located within gene promoters, with the exception of the constitutive group (35%; Figure 4D). Gene ontology analysis showed that temporally patterned DHSs are highly enriched near genes of specific classes, commensurate with the developmental functions of those genes. Early clusters 1, 2, and 3 are generally associated with genes involved in stem cell maintenance, neuron generation, and gliogenesis (Additional file 10: Figure S10), reflecting the fact that neuronal production peaks in the retina at birth. Cluster E4 DHSs are primarily active at P0, but have some activity at P7, and are enriched near genes associated with synaptogenesis (that is, ‘dendritic spine development’). Mid-stage clusters M1 and M2 are generally associated with many of the same genes in the early clusters but also include the later processes of ‘axon extension’ (Additional file 10: Figure S10), in agreement with the extensive neuronal differentiation and synapse formation that occurs at P7 [48]. Late clusters 1, 2, and 4 are generally associated with perception of light and photoreceptor maintenance (Additional file 10: Figure S10), reflecting the fact that photoreceptors comprise approximately 80% of the adult mouse retina.

As discussed in the previous section, *Crx* and *Nrl* are two transcription factors that play a role in photoreceptor differentiation, and their binding sites coincide extensively with retina-specific DHSs. Using the CRX and NRL ChIP-seq data (see above), we find that the majority of CRX and NRL binding occurs in DHSs active in late-enriched stages of retinal development (66% and 60%, respectively; clusters L1 to L4, Figure 4D). This corresponds to known patterns of expression for these genes ([40, 41]; Additional file 11: Figure S11). However, there are some CRX and NRL binding sites present in DHSs active at all ages (30% and 34%, respectively). This suggests that some potential regulatory elements for photoreceptors are accessible even early in their development (P0) while others become accessible as the cells mature; however, it is also important to note that the number of rods doubles between P0 and P7, and so this pattern might also reflect this change in cell number.

Given the enrichment of temporally patterned retina DHSs near specific classes of genes, we next sought to identify which transcription factors (TFs) could be controlling these patterns by analyzing TF binding motifs within the *k*-means-clustered DHSs. The full list of factors (Additional file 12: Figure S12) reveals significant enrichment (*P* < 0.01) of motifs for various TFs known to be involved in retinal development and neurogenesis. The cluster enrichment of a selected subgroup of TFs important for retinal development is displayed in Figure 4E. Importantly, the early clusters (especially E1 and E2) are enriched for motifs of TFs that are active in the developing retina (for example, *Lhx2*, *Pou3f2*) [49, 50], whereas the late clusters (L1, L2, and L4) are enriched for motifs of factors vital for mature retinal function (for example, *Otx2*, *Crx*) [45]. When we instead analyzed retina-specific DHSs, the motifs of 22 TFs are significantly enriched (*P* < 0.01; Additional file 13: Table S4), some of which have important functions in the retina (for example, *Rax*, *Otx2*, *Crx*). Interestingly, many motifs enriched in temporally dynamic as well as retina-specific DHSs are recognized by TFs with as yet unexplored roles in retinal development.

Overall, these results show that temporal dynamics of specific developmental processes are reflected in temporal changes in chromatin accessibility surrounding genes involved in these processes. Furthermore, examination of TF motifs within temporally patterned DHSs can be used to identify the transcription factors that regulate these processes.

### Functional analysis of temporally dynamic DHSs surrounding Otx2

**Figure 5:**
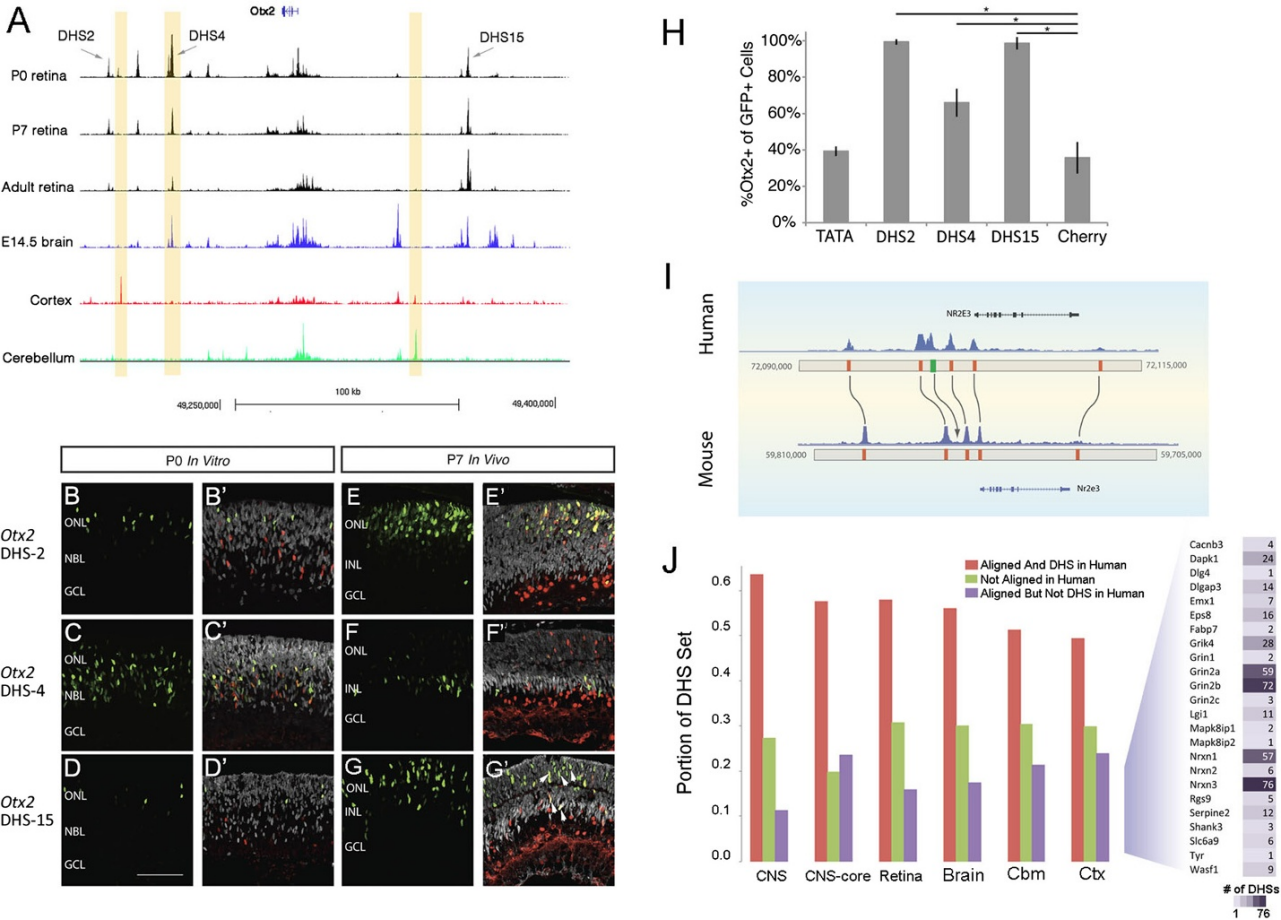
**Functional testing of retinal DHSs near** ***Otx2*** **, and the conservation between mouse and human. (A)** The DNase I cleavage landscape is shown surrounding the *Otx2* gene for P0, P7, and adult (Ad) retina to highlight developmental DHS dynamics. Selected *Otx2* DHSs labeled with arrows. Tan shading highlights DHSs differentially active in the retina, cerebellum, and cerebral cortex. **(B-G’)** Panels show representative images of expression from *Otx2* DHS reporter constructs (green) co-immunostained for transfection control plasmid (CHERRY, red) and endogenous OTX2 (white). Arrows **(G’)** highlight five example OTX2+ GFP+ co-expressing cells. Left two columns show expression from indicated constructs in electroporated P0 retinal explants cultured 24 h *in vitro* **(B-D’)** (*N* = 3). Right two columns show expression from indicated constructs in retinas electroporated *in vivo* at P0 and harvested at P7 **(E-G’)** (*N* = 2 to 5). ONL, outer nuclear layer; NBL, neuroblastic layer; GCL, ganglion cell layer; INL, inner nuclear layer. Scale bar = 200 μm. **(H)** Quantification of electroporation data showing that nearly 100% of the cells expressing *Otx2* DHS-2 or *Otx2* DHS-15 also express OTX2 in P0 retina. (*N* = 3) *P < 0.01; error bars ± SD. **(I)** Alignment of human and mouse genomes showing the sequence (orange rectangles) conservation of DHSs surrounding the *Nr2e3* gene, important in rod photoreceptor gene expression. **(J)** Comparison of functional and/or sequence conservation of human and mouse DHSs for the CNS (set of DHSs common to mature retina and brain), CNS-core (DHSs only active in the CNS), retina, cerebellum, adult whole brain (Brain), and cerebral cortex (Cortex). Red, DHS in mouse, orthologous region is a DHS in human; purple, DHS in mouse, orthologous region is not a DHS in human; green, DHS in mouse, no orthologous sequence in human. Right blow-up: genes associated with mouse cortex DHSs that have orthologous sequence but no activity in the human genome; numbers of DHSs near respective genes, expressed as the intensity of colored cells.

We chose to further examine the *Otx2* locus due to the critical role for this gene in retinal development and the enrichment of its binding motif in retina-specific DHSs [51]. The *Otx2* locus contains many developmentally dynamic DHSs, which show either an increase or a decrease in accessibility across stages and were assigned to early or late clusters in our *k*-means analysis. The DHS map (Figure 5A) reveals a previously identified distal *Otx2* enhancer (FM1 [52]) and other cis-regulatory elements [53], in addition to several potentially novel cis-regulatory elements. We selected 17 regions on the basis of DNase I accessibility and/or evolutionary sequence conservation for further study. Most striking are *Otx2* DHS-4 (approximately 53 kb downstream of *Otx2*), which is highly active in P0 retina but has decreased activity in the P7 and adult retina, and *Otx2* DHS-15 (approximately 79 kb upstream of *Otx2*), which shows the opposite pattern (Figure 5A). Chromatin immunoprecipitation for the transcriptional co-activator P300, which has been shown to localize with active enhancer elements [10], showed that several of the *Otx2* DHSs were positive (>0.3% input, determined by *Irbp* positive control) for P300 binding (Additional file 14: Figure S14F).

To determine whether the *Otx2* DHSs function as transcriptional enhancers, we tested these elements for their ability to drive expression of a green fluorescent protein (GFP) reporter construct in retinal tissue. Each DHS with a P300 ChIP signal above the positive control (*Otx2* DHS #1, 2, 4, 7, 8, 10, and 15) was cloned into a minimal promoter vector and was electroporated on the day of birth along with a transfection control plasmid constitutively expressing nuclear-CHERRY. We assayed reporter expression after 1 day (*in vitro*) or after 7 days (*in vivo*); the TATA box minimal promoter was used as a negative control (Figure 5B,C,D,E,F,G; Additional file 14: Figure S14A,B,G). *Otx2* DHS #1, 7, 8, and 10 showed no functional activity in driving reporter expression in the retina (data not shown). However, we found that several *Otx2* DHSs robustly drive expression of the GFP reporter in the retina. *Otx2* DHS-4, for example, drives GFP expression primarily in the progenitor zone (middle and outer retina) at P0 (Figure 5C), while the GFP expression from the *Otx2* DHS-2 construct is concentrated in the outer retina where nascent photoreceptors reside (Figure 5B). *Otx2* DHS-2 and *Otx2* DHS-15 GFP+ cells are nearly 100% OTX2+ at P0 (Figure 5H), though for *Otx2* DHS-4, the percentage of OTX2+ cells was lower (Figure 5H). In the P7 retina, expression driven by *Otx2* DHS-4 is reduced (Figure 5F), and the GFP+ cells are typically found in the inner nuclear layer, co-localized with OTX2+ bipolar cells. At this developmental time, a greater fraction of the *Otx2* DHS-4 GFP+ cells are OTX2+ (94%) (Additional file 14: Figure S14C). Nearly 100% of cells expressing GFP driven by *Otx2* DHSs 2 and 15 co-express OTX2 at both P0 and P7 (Figure 5B,D,E,G,H; Additional file 14: Figure S14C). These results together demonstrate that DHS analysis can identify new enhancers active in distinct cell populations in developing and mature retina.

### Shared DHS activity between mouse and human varies across brain regions

The ENCODE Consortium has generated a catalog of potential functional elements in the human genome and more recently the mouse genome (ENCODE Consortium 2012 [16]). Between one half and two thirds of these cis-regulatory elements are conserved between mouse and human (for example, 61% of mouse DHSs have identifiable orthologous sequences and are also DHSs in human; Figure 5I,J). However, despite high sequence conservation, many elements no longer have an orthologous chromatin signature, suggesting that they have diverged functionally. Interestingly, enhancers of developmental regulatory genes are among those with the greatest amount of both functional and sequence conservation between mouse and human [16].

We asked whether functional conservation of CNS regulatory elements between mouse and human varies by brain region. Vierstra *et al.*[16] found that the median conservation of DHS activity across all tissues analyzed between mouse and human is 48%, with a range of 38.2% to 60.3%, referred to as ‘shared’ DHSs. The percentage of shared DHSs is relatively high in the CNS, with over 60% of DHSs identified in this study shared between mouse and human (Figure 5I,J). CNS-core DHSs contain a greater percentage that are unique to mouse than the overall set of CNS DHSs (Figure 5J, Additional file 15: Table S5 and Additional file 16: Table S6).

Comparing the proportions of shared DHSs within CNS regions revealed significant differences among their distributions (Figure 5J; Additional file 15: Table S5). The retina and cerebellum have a somewhat lower percentage of shared DHSs than the overall CNS. However, the cerebral cortex has the lowest percentage of shared DHSs of the regions analyzed, possibly reflecting the greater extent of divergence of this structure between mice and men. We subjected mouse cerebral cortex DHSs that have conserved sequence but are not DHSs in human to GREAT analysis; among the highest scoring categories (either by gene number or *P* value) are genes associated with NMDA receptors and their regulation. Figure 5J, right blow-up, shows the genes in this category with the associated number of mouse DHSs that have lost activity in human. The genes with the greatest number of unshared DHSs between mouse and human are the subunits of the NMDA receptor (*Grin2a* and *Grin2b*), critical for neuroplasticity and memory, and the neurexins (*Nrxn1* and *Nrxn3*), highly differentially spliced genes involved in synapse formation [54, 55]. These results suggest that re-wiring of the cis-regulatory elements controlling genes associated with neural plasticity and synaptogenesis may accompany evolutionary changes in brain function.

## Discussion

Defining the gene regulatory networks that control the vast cellular diversity and connectivity in the mammalian CNS presents a significant challenge for traditional approaches. The development of DNase I hypersensitivity mapping at the genome-wide scale has provided new approaches to characterize CRMs and the transcription factors that recognize them [22, 23]. We applied this technique to the mouse CNS; by sampling different brain regions as well as developmental stages, we were able to identify CRMs with regional, temporal, and cell type specificity (even for minor cell populations) in the CNS. A similar study using H3K27ac ChIP-seq for mouse cerebral cortex, heart, and liver demonstrated the power of comparing enhancer activity across developmental transitions and tissues [15]. We found that similar conclusions can be applied to the different regions of the central nervous system and potentially to individual cell types. Moreover, the sensitivity of DNase I hypersensitivity mapping has allowed us to identify nearly ten times more putative cis-regulatory regions in the brain and retina than were identified with previous studies using ChIP.

By comparing the DHSs from the brain and retina, we were able to delineate a core set of DHSs common to the CNS, the majority of which are shared between mouse and human, representing the accessible chromatin of the brain and retina. Not surprisingly, the core set is enriched for DHSs near neural genes, like neurotransmitter receptors and ion channels, and provides a new resource for studies of their regulation. In addition, there appear to be many regulatory elements that potentially regulate expression of neural-expressed genes involved in brain disorders (for example, *Parkin2*, *Lingo1*, *Dscam*, *Msra*). In light of recent evidence that disease-associated polymorphisms, identified by GWAS studies, are concentrated in DHSs [56], the core-DHS regulome provides thousands of new candidate regions for potential disease-causing mutations.

In addition to the core set of DHSs common to all regions of the CNS, when we compared different regions of the CNS, we were able to identify DHSs unique to each region. Genes expressed in many regions of the CNS, like *Nefl*, show different patterns of DNase I hypersensitivity depending on the CNS region, and these differences allow the identification of potential enhancers that regulate expression in specific CNS regions and potentially even in specific cell types. For example, of the DHSs near the *Otx2* locus that we tested experimentally, we found that there was selectivity for both developmental stage (developing vs mature) and retinal cell type (photoreceptor vs bipolar cell). It is likely that the same will hold true for other brain regions, and thus, the DHSs we have identified could potentially be involved in regulating gene expression in subsets of neurons within these regions. Further testing of candidate elements in transgenic assays will be needed to validate this possibility.

It is interesting that we are able to detect DNase I-seq peaks at the promoters and enhancers of genes expressed in only a subset of the total cell population within a given region of the CNS. There is a good correlation between the chromatin accessibility and the expression of a gene in a particular tissue, and in this report, we find that this correlation extends to regional differences within the CNS. The data also suggest that this correlation might extend to the level of specific cell types within the CNS regions. However, at this point, we do not know whether the DHSs near genes expressed in minority neuronal populations (for example, *Opn1sw*) are only present in these cells or are present in other cells in the population that do not express these genes. Further experiments involving isolation of individual cell populations of the brain or retina will be needed to determine the specificity of these DHSs.

Functional analyses of putative cis-regulatory regions, both *in vitro* and *in vivo*, of a key CNS gene validates this approach for the identification of enhancers for specific CNS regions and developmental stages. Several previous studies have used comparative and/or epigenetic approaches to identify CRMs in both developing and mature tissues, including the CNS [2, 5, 7, 10, 11, 14, 28, 29, 57–59]. With the DNase I signal alone, we were able to predict elements that drive expression in the developing brain in the VISTA Browser to a similar degree as a recent H3K27ac ChIP-seq study [15]. Combining the DNase-seq data with other epigenetic markers of enhancers, such as H3K27ac and P300, should continue to refine their predictive power. Moreover, since DNase-seq also identifies promoters, insulators, and virtually every class of active regulatory element, this technique provides a more comprehensive view of the epigenome, although this view is inherently non-specific to the nature and function of identified regulatory elements.

Combined with transcription factor motif identification, DNase I hypersensitivity mapping can also delineate transcriptional networks in the developing and mature brain. By comparing DHSs from three different ages of retinal development, we were able to identify stage-specific transcription factor binding motifs for known developmental regulators enriched in retinal samples of each age and generate a list of potential transcriptional regulators relevant to distinct developmental processes and mature gene regulation. Recent studies have shown that DHS motif analysis, along with digital footprints, can be used to generate potential regulatory networks directly [60]. The transcription factor networks can be generated from motif analysis within DHSs and digital genomic footprinting to validate ChIP-seq data and to generate *de novo* predictions about potential transcriptional regulators of specific genes.

By comparing the core DHS set from mouse CNS with DHSs of human tissues, we found that the conservation of CNS DHSs between mouse and human is only about 60%. This is close to that observed across all tissues by Virestra *et al.*[16] and reflects the rapidly evolving cis-regulatory landscape revealed by DNase-seq analysis and other approaches [15]. There are region-specific differences in DHS divergence between these species, with the cerebral cortex having the lowest percentage of shared DHSs of the regions analyzed. This might be due to the greater extent of divergence of this structure between mice and men than other CNS regions, like the cerebellum and retina. Although this conclusion is speculative at this time, our analysis suggests that divergence in cis-regulatory elements near genes associated with neural plasticity and synaptogenesis might be important in brain evolution. Focusing studies of evolution to the sequence content within DHSs may provide a more efficient approach to studying the evolution of gene regulation across species.

## Conclusions

The complexity of the CNS is generated in part through transcriptional regulation of gene expression, and the data in this report provide an additional approach to the identification of genomic regions and the elucidation of the cis-regulatory mechanisms involved in this process.

## Methods

### Animals

C57BL/6 J mice (Jackson Laboratory, Bar Harbor, ME, USA) were used for all experiments, housed in the University of Washington Department of Comparative Medicine. All experiments were carried out according to approved protocols by the University of Washington Institutional Animal Care and Use Committee (IACUC) protocol #2448-08.

### Nuclei isolation

Retinal and brain tissue was dissected (minced into approximately 2-mm^3^ pieces) and suspended in 3 mL homogenization buffer (20 mM tricine, 25 mM D-sucrose, 15 mM NaCl, 60 mM KCl, 2 mM MgCl_2_, 0.5 mM spermidine, pH 7.8). Tissue was Dounce-homogenized with 5 to 10 strokes with loose, type A pestle (brain tissues) or with 5 and 25 strokes of loose and tight pestle, respectively (retina tissues), followed by filtration through a 100-μm filter. Nuclei suspension was then cryopreserved by addition of DMSO to 10%, controlled freeze to -80°C, and subsequently stored in liquid nitrogen. After thaw and before DNase I treatment, buffer was exchanged with 15 mL sucrose buffer (10 mM Tris-HCl, 250 mM D-sucrose, 1 mM MgCl_2_, pH 7.5), and nuclei were collected by centrifugation (600 *g*, 10 min, 4°C) and resuspended in 10 mL fresh sucrose buffer. Nuclei were passed through a 20-μm filter and centrifuged (600 *g*, 10 min, 4°C). The pelleted nuclei were washed with 10 mL of buffer A (15 mM Tris-HCl, 15 mM NaCl, 60 mM KCl, 1 mM EDTA, 0.5 mM EGTA, 0.5 mM spermidine) and resuspended to two million nuclei per mL.

### DNase I treatment

Nuclei were incubated at 37°C for 3 min with limiting concentrations of DNase I enzyme in buffer A supplemented with Ca^2+^. The reaction was terminated with an equal volume of stop buffer (50 mM Tris-HCl, 100 mM NaCl, 0.1% SDS, 100 mM EDTA, 1 mM spermidine, 0.5 spermine, pH 8.0) and subsequently treated with proteinase K and RNase A at 55°C. Small (<750 bp) DNA fragments were recovered via sucrose ultracentrifugation and subsequently end-repaired and ligated with Illumina-compatible adaptors. A detailed description of the mapping of DNase I-hypersensitive sites is available in reference [18].

### Sequence alignment and DHS scanning algorithm

Sequence reads (36 bp) were mapped to the human (GRCh37) and mouse (NCBI37) genomes using bowtie (v 0.12.7) [61]. Sequencing reads varied between samples (Additional file 1: Table S1). To account for this variability, we down-sampled each mouse tissue sample to 25 million reads (random sampling, no replacement) and subsequent datasets were used for DNase I peak and hotspot calling. Reads were summed within 150-bp windows in 20-bp steps and normalized to the number of reads per tissue sample dataset.

The Hotspot algorithm (reference [19], detailed description of calculations can be found at http://www.uwencode.org/proj/hotspot/) was used to detect distinct regions of chromatin accessibility. Localized enrichments of sequence tags are identified based on a binomial distribution model computed against a local background model surrounding each tag. Regions of enrichment are termed hotspots and are further internally scanned for the local maxima; 150-bp windows around the local maxima are called as peaks. To generate a false discovery rate (FDR 1% for all datasets), simulated datasets are generated based on random reads at equal sequencing depth to each sample dataset and the simulated data was subsequently scanned for hotspots to determine an estimate FDR. Dataset quality is also measured using a SPOT (signal portion of tags) score defined as the percentage of tags that fall into hotspots (http://www.uwencode.org/proj/hotspot/).

### Global analysis of DHS landscape

The mouse Ensembl65 genomic coordinates were used as the basis for this analysis, and BEDOPS [62] was used to determine overlap between DHSs and genomic features. CRX ChIP-seq peaks were determined by the intersection of peaks from CRX ChIP-seq replicates 1 and 2 using BEDOPS.

### *k*-means clustering analysis

A ‘master list’ of 197,962 non-redundant, non-overlapping retinal DHSs from all three stages was created as previously described [22]. The maximum read-count-normalized DNase I tag density was then determined for each master list DHS in each sample. Each sample’s tag density values were divided by the sample’s SPOT score, a quality metric for DNase-seq library complexity, to control for differences in sample quality. Tag density values were then transformed by log10 (density + 1) and row-normalized across stages at each DHS to set the maximum density value to 10. The DHSs were then subjected to *k*-means clustering to create 12 groups containing DHSs with similar temporal activity across the three sampled developmental stages.

### Functional annotation of DHSs

DHSs within a region 1 kb upstream of Ensembl65-annotated transcription start sites were classified as promoter DHSs. CRX and NRL binding regions were obtained from ChIP-seq data in [42] and [43]. Master list DHSs used for *k*-means clustering were considered occupied by CRX and/or NRL if the peak calls for these two factors overlapped a DHS by at least 75 bp. Of 5,724 CRX binding sites, 178 failed to overlap a DHS and 224 overlapped more than one DHS; the latter were not considered for calling CRX-occupied DHSs. Of 7,303 NRL binding sites, 1,707 fail to overlap a DHS and 260 overlap more than one DHS. Retina-specific DHSs were independently determined in a study of the human and mouse regulatory landscapes [16]; the master-list DHSs in this study were considered to be retina-specific if they overlapped a DHS called by Vierstra *et al.*[16] by at least 25 bp. The list of retina-specific DHSs was generated as described in Vierstra *et al.*[16].

### Motif enrichment in DHSs

Putative transcription factor binding sites were identified by scanning the entire mouse genome for consensus sequences using the FIMO tool from the MEME Suite (version 4.6) [34] with default parameters, using motif models curated from TRANSFAC (version 11) [63], JASPAR [64], and UniProbe [65]. Each motif model was linked to a transcription factor gene, allowing for redundancies in the motif databases; multiple TFs were allowed to be paired with the same motif, and many TFs were represented by multiple motif models. We then determined the number of DHSs containing a motif match (FIMO *P* value <10e - 4) for each TF and used a cumulative hypergeometric distribution to calculate a *P* value for the enrichment of that TF’s motifs within DHSs assigned to specific *k*-means clusters (or retina-specific DHSs) compared to the overall prevalence of its binding sites in master-list DHSs. *P* values were corrected for multiple testing using the Bonferroni method.

### Electroporations

Reporter plasmids contain the experimental DHS sequence immediately upstream of a minimal promoter containing TATA box driving nuclear GFP; control plasmids contain the *Ef1a*-promoter driving nuclear CHERRY red fluorescent protein. Retinal explants (dissected retina tissue cultured *in vitro*) were electroporated in PBS with 2 μL DNA (2.33 g/μL ECR-GFP plus 1 g/μL mCherry control plasmid) using an ECM830 Square Wave Electroporation System (BTX Harvard Apparatus, Hollisto, MA, USA) with the following settings: 35 V, 5 pulses, 50 ms/pulse. Retinas were then cultured in six-well tissue culture plates with 1 mL of Neurobasal media, with 1% FBS (Clontech, Mountain View, CA, USA), 1 mM l-glutamine (Invitrogen, Carlsbad, CA, USA), N2 (Invitrogen), and 1% penicillin-streptomycin (Invitrogen) for 24 to 36 h. Brains were dissected from mice and sliced with a McIlwain Tissue Chopper (Vibratome 800, Ted Pella, Inc., Redding, CA, USA) set to 300-μm sections. Individual brain sections were electroporated as above and cultured on 0.4-μm Millicell cell culture inserts (Millipore PICM03050, Millipore, Billerica, MA, USA) in six-well tissue culture plates containing 1 mL neurobasal media (above) for 36 h. For *in vivo* electroporations, P0 mice were anesthetized on ice and 1 μL DNA (2.7 g/μL ECR-GFP and 0.3 g/μL mCherry control plasmid) was injected into the vitreous of the eye (syringe: Hamilton 7635-01; needle: 32-gauge Hamilton 7803-04, Hamilton, Reno, NV, USA). Electroporation was performed with head paddles connected to the ECM830 Square Wave Electroporation System with the following settings: 90 V, 5 pulses, 50 ms/pulse, 950-ms intervals. Mice were revived at 37°C and retinas were harvested 7 days later.

### Immunohistochemistry and microscopy

Retinas or brain explants were fixed with 2% paraformaldehyde. Retinas were cryoprotected in 30% sucrose/PBS at 4°C overnight, embedded in OCT compound (Sakura Finetek, Torrance, CA, USA), and sectioned at 12 μm using a cryostat. Immunohistochemistry (IHC) was carried out using chicken anti-GFP (1:500, ab13970, Abcam, Cambridge, UK), rabbit anti-RFP (1:500, Clontech #632496), biotinylated anti-Otx2 (1:100, BAF1979, R&D Systems, Minneapolis, MN, USA). All secondary antibodies were from Life Technologies (Carlsbad, CA, USA) or Jackson ImmunoResearch (West Grove, PA, USA) and used at 1:500. Imaging was performed using an Olympus FluoView confocal microscope (Olympus Corporation, Tokyo, Japan).

### Chromatin immunoprecipitation

Otx2 ChIP: P0 and adult retinas were digested with papain into single cell suspension and fixed with 0.5% formaldehyde, 10 min, rotating at room temperature (RT). Sonication (Fisher Scientific, Waltham, MA, USA) was performed: 12 pulses of 100 J, 35 amplitude with a 45-s offset at 4°C. Immunoprecipitation performed with 20 μL anti-rabbit IgG magnetic beads (Invitrogen, #112-03D) and 2 μg goat anti-hOTX2 antibody (R&D Systems BAF1979) or 2 μg goat IgG (R&D Systems AB-108-C) against chromatin from 1e6 cells (P0) per IP according to LowCell# ChIP Kit (Diagenode, Liège, Belgium). DNA sequences were quantified with Bio-Rad CFX96 thermocycler using SsoFast EvaGreen Supermix (Bio-Rad, Hercules, CA, USA) according to the manufacturer’s instructions. All values were expressed as a percentage of input DNA averaged from at least three biologically independent experiments.

## Authors’ information

MW is a graduate student in Molecular and Cellular Biology at the University of Washington. JB was a post-doctoral fellow in the Department of Biological Structure at the University of Washington and is now an assistant professor in Ophthalmology at the University of Colorado, Denver. AL was a post-doctoral fellow in the Department of Biological Structure at the University of Washington and is now an assistant professor at the University of California, Davis. JV and KS are post-doctoral fellows in the Department of Genome Sciences at UW. RT, PS, RS, and TC are scientists in the Department of Genome Sciences at UW. RH is a research associate professor of Medical Genetics at UW. MB is an associate professor of Pediatrics at the University of Washington Medical School and a member of the Fred Hutchinson Cancer Research Center. JS is a professor of Genome Sciences at the University of Washington. TR is a professor of Biological Structure at the University of Washington.

## Electronic supplementary material

Additional file 1: Table S1: Sequencing and accessibility information of DNase I hypersensitivity sequencing of samples analyzed in this manuscript. Basic tissue, sequencing and data accessibility information in tabular format. (DOCX 118 KB)

Additional file 2: Figure S2: Genomic partition, Gene Ontology Molecular Function enrichment, and Gene Ontology analysis. (A) Genomic partition of the cortex, cerebellum, and retina DHSs. Distribution of DHSs present in mature cerebral cortex and cerebellum brain regions and mature retina relative to genomic features. (B) Gene Ontology Molecular Function enrichment of CNS-core DHSs. (C) Gene Ontology analysis from GREAT, Molecular Function category, of the CNS-core set of DHSs showing enrichment near neuronal genes. (JPEG 262 KB)

Additional file 3: Table S2: Genomic intervals for ‘CNS core regulome’ DNase I-hypersensitive sites. Genomic interval file in tabular format for DHSs common to CNS, but not other tissues. (TXT 152 KB)

Additional file 4: Figure S4: DNase I hypersensitivity corresponds to enhancer regions identified in previous studies and confirmed in transgenic mice for the VISTA Browser project [30]. Three different enhancers with their expression patterns are shown at the right in transgenic mice and as a tan-shaded region in the UCSC browser tracks. The P300 and H3K27ac ChIP-seq peaks from previous studies are labeled as in Figure 1D. The mm871 enhancer (tan shaded) shows overlap with the DNase I peak, the P300 ChIP-seq peaks, and the H3K27ac peak, whereas the other two enhancers show a DNase I hotspot and two of the three other marks. (JPEG 148 KB)

Additional file 5: Figure S5: Sets of enriched DHSs between CNS tissues and CentriMo analysis. (A) The sets of DHSs enriched for the cerebral cortex, cerebellum, or retina were analyzed with the MEME suite (DREME and CentriMo), and we found a distinct pattern of enrichment for transcription factor motifs in the different sets. EGR1 sites and bHLH transcription factor sites were highly enriched in the cerebral cortex, whereas Crx sites were over-represented in DHSs from the retina. (B) CentriMo analysis for EGR1 and E-box sites in cerebral cortical DHSs shows enrichment near the central regions of these DHSs for the over-represented transcription factor motifs, consistent with their role as enhancers. Below: GO enrichment terms associated with EGR1 and E-box sites in the cortex. (JPEG 284 KB)

Additional file 6: Figure S6: DNase I hypersensitivity at the promoters of cell type-specific genes. DNase I landscape (cerebral cortex, Ctx (red); cerebellum, Cbm (green)) surrounding the gene bodies of (A) *Etv1* and (B) *Kcnn2* and accompanying *in situ* data (A’-A”’, B’-B”). Black arrows point to cell layers with positive (dark purple) *in situ* signal. *In situ* data from 2014 Allen Institute for Brain Science. Available from http://mouse.brain-map.org/[66]. (C) DNase I landscape from the P0 retina for cell type-specific genes: *Opn4* (ganglion cells), *Th* (amacrine cells), *Gnat2* and *Opn1mw* (cone photoreceptors). Tan box indicates DHS at the promoter of each gene. (PDF 4 MB)

Additional file 7: Table S3: Genomic intervals for retinal specific DNase I-hypersensitive sites. Genomic interval file in tabular format for DHSs unique to the mouse retina, but not other tissues. (TXT 69 KB)

Additional file 8: Figure S8: Gene ontology enrichment of retinal specific DHSs. Gene ontology (A) biological process and (B) disease ontology categories for genes associated with retina-specific DHSs as determined by GREAT analysis. (PDF 3 MB)

Additional file 9: Figure S9: (Related to Figure 4A,B) RNA-seq landscape for P2 and P21 retina surrounding two progenitor genes expressed in the early retina: *Neurog2* and *Olig2*, and three photoreceptor genes expressed in the mature retina: *Rho* and *Guca1a*/*b*. RNA-seq data previously generated by [45]. (JPEG 213 KB)

Additional file 10: Figure S10: Gene ontology analysis of genes near *k*-means-clustered DHSs from retina tissue. Gene ontology (biological process) categories for genes associated with DHSs within each *k*-means temporal cluster of retinal DHSs (P0, P7, and adult retina) as determined by GREAT analysis. E, early clusters; M, mid-clusters; L, late clusters; O, other cluster groups; C, constitutive cluster group. (JPEG 579 KB)

Additional file 11: Figure S11: (Related to Figure 4D,E) The RNA-seq landscape for P2 and P21 retina and the DNase I landscape for P0, P7, and 8-week adult (8w) retina surrounding two retinal development and differentiation genes: *Nrl* and *Crx*. RNA-seq data previously generated by [45]. (JPEG 137 KB)

Additional file 12: Figure S12: Transcription factor binding motif enrichment within temporal clusters of total retinal DHSs. Transcription factor binding motif enrichment (-log(*P* value)) indicated as color intensity for each transcription factor (rows) within each temporal cluster group (columns) from P0, P7, and adult stages of mouse retina. E, early clusters; M, mid-clusters; L, late clusters; O, other cluster groups; C, constitutive cluster group. (JPEG 694 KB)

Additional file 13: Table S4: Retina-specific DHSs: transcription factor binding motif enrichment. Transcription factor binding motif enrichment from DHSs unique to the mouse retina (not present in other mouse tissues). (DOCX 64 KB)

Additional file 14: Figure S14.: *Otx2* DHS reporter expression and transcription factor binding. (A, B) Panels show representative images of expression from empty minimal reporter constructs (TATA) with no *Otx2* DHS insert (green) co-immunostained for transfection control plasmid (CHERRY, red) and endogenous OTX2 (white). (A) Expression from TATA in electroporated P0 retinal explants cultured 24 h *in vitro* (*N* = 3). (B) Expression from TATA in retinas electroporated *in vivo* at P0 and harvested at P7 (*N* = 3). ONL, outer nuclear layer; NBL, neuroblastic layer; GCL, ganglion cell layer; INL, inner nuclear layer. (C) Quantification of the percentage of GFP+ cells that co-express OTX2+ for *Otx2* DHSs, non-specific control plasmid (TATA), and transfection control (CHERRY) in P7 retina *in vivo* (*N* = 2 to 5). *P < 0.01; error bars ± SD. (D, E) Chromatin immunoprecipitation for OTX2 or IgG control from P0 (D) and Adult (E) whole retina tissue shown as a percentage of input DNA. Assayed regions are *Otx2* DHSs 2, 4, 12, and 15 with the *MyoD* promoter serving as a negative control. *N* = 3 ± S.D. *P < 0.05; **P < 0.01; ***P < 0.001, ****P < 0.0001. Error bars ± SD. (F) Chromatin immunoprecipitation for P300 or IgG control shown as a percentage of input DNA for *Otx2* DHSs, the *Otx2* promoter (pOtx2), previously described enhancers (FM1, FM2, AN), and a positive control promoter (pIrbp). *N* = 2 to 4 ± S.D. (G) Inset from Figure 5G showing separated color channels and the colocalization of expression from *Otx2* DHS #15 reporter construct (green) co-immunostained for transfection control plasmid (CHERRY, red) and endogenous OTX2 (white). Arrows indicate examples of triple positive labeled cells. (PDF 3 MB)

Additional file 15: Table S5: *P* values resulting from pairwise chi-squared on DHS distribution into conservation categories. Related to Figure 5J. (DOCX 43 KB)

Additional file 16: Table S6: Mouse DHS alignment and conservation with human DHSs. The numbers of mouse DHSs falling into each category of alignment and DHS conservation in humans. (DOCX 77 KB)

## Abbreviations

CNS: Central nervous system
CRM: Cis-regulatory module
DHS: DNase I-hypersensitive site
P0: Post-natal day 0
P7: Post-natal day 7
TF: Transcription factor
TSS: Transcription start site.
