# Supplementary material for: DNase I hypersensitivity analysis of the mouse brain and retina identifies region-specific regulatory elements
Source: Epigenetics Chromatin. 2015 Feb 28;8:8. doi: 10.1186/1756-8935-8-8 (PMC4429822; doi:10.1186/1756-8935-8-8)
Supplement: Supplementary file 1 — Additional file 1: Table S1: Sequencing and accessibility information of DNase I hypersensitivity sequencing of samples analyzed in this manuscript. Basic tissue, sequencing and data accessibility information in tabular format. (DOCX 118 KB) [file 13072_2014_358_MOESM1_ESM.docx]

Supplemental Table S1: Sequencing and Accessibility Information of DNaseI hypersensitivity sequencing of samples analyzed in this manuscript.

| Species | Tissue | Age | Tags | SPOT score | Peaks 1% FDR | GEO Accesion |
| --- | --- | --- | --- | --- | --- | --- |
| Mouse | Retina | Post-Natal day 0 | 35,959,145 | 0.484 | 134,712 | GSM1014188 |
| Mouse | Retina | Post-Natal day 7 | 26,973,855 | 0.418 | 109,073 | GSM1014198 |
| Mouse | Retina | 8-week Adult | 28,252,986 | 0.323 | 100,570 | GSM1014175 |
| Mouse | Whole Brain | Embryonic day 14.5 | 25,249,691 | 0.483 | 167,715 | GSM1014197 |
| Mouse | Whole Brain | 8-week Adult | 36,079,464 | 0.689 | 224,978 | GSM1014151 |
| Mouse | Cerebellum | 8-week Adult | 21,690,965 | 0.397 | 106,514 | GSM1014164 |
| Mouse | Cerebrum | 8-week Adult | 43,834,852 | 0.437 | 242,095 | GSM1014168 |
| Human | fetal Retina | day 87 | 34,942,536 | 0.49 | 209,105 | submitted |
| Human | fetal Retina | day 103 | 46,713,047 | 0.41 | 343,803 | submitted |
| Human | fetal Retina | day 125 | 43,011,675 | 0.7 | 184,852 | submitted |
| Human | fetal Brain | day 117 | 25,016,845 | 0.59 | 195,888 | GSM595920 |
| Human | fetal Brain | day 85 | 23,608,044 | 0.405 | 184,688 | GSM595923 |
| Human | fetal Brain | day 96 | 20,887,542 | 0.555 | 177,090 | GSM595928 |
| Human | fetal Brain | day 112 | 34,671,585 | 0.386 | 191,014 | GSM665804 |
| Human | fetal Brain | day 142 | 34,462,349 | 0.434 | 167,734 | GSM665819 |
| Human | fetal Brain | day 101 | 30,786,898 | 0.416 | 216,130 | GSM878650 |
| Human | fetal Brain | day 104 | 34,982,802 | 0.584 | 191,232 | GSM878651 |
| Human | fetal Brain | day 109 | 24,905,787 | 0.436 | 178,475 | GSM878652 |
| Human | fetal Brain | day 105 | 34,978,828 | 0.49 | 204,455 | GSM1027328 |
| Human | fetal Brain | day 122 | 25,472,137 | 0.72 | 182,501 | GSM530651 |
