# Supplementary figures and images for: DNase I hypersensitivity analysis of the mouse brain and retina identifies region-specific regulatory elements
Source: Epigenetics Chromatin. 2015 Feb 28;8:8. doi: 10.1186/1756-8935-8-8 (PMC4429822; doi:10.1186/1756-8935-8-8)

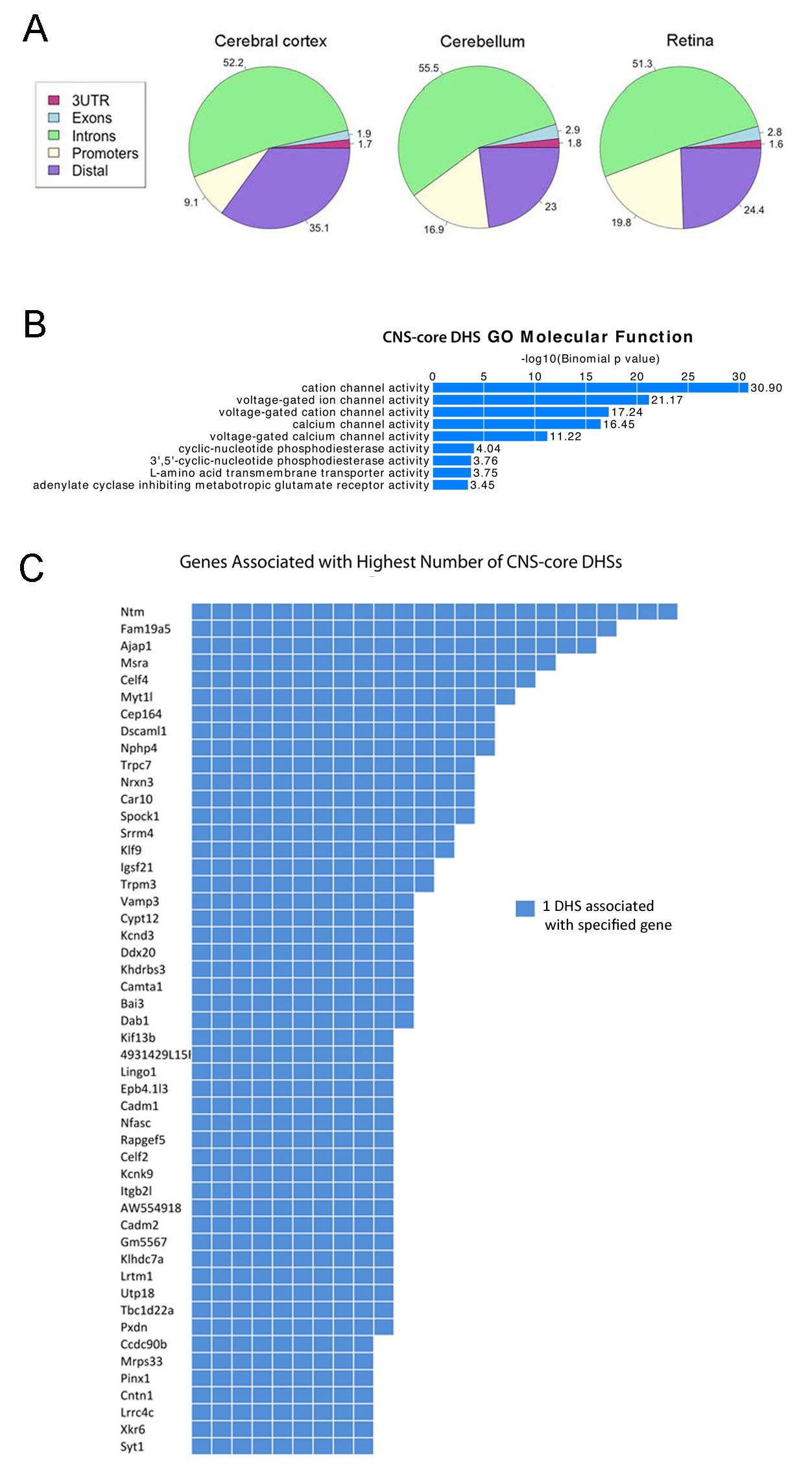

Supplement: Supplementary file 2 — Additional file 2: Figure S2: Genomic partition, Gene Ontology Molecular Function enrichment, and Gene Ontology analysis. (A) Genomic partition of the cortex, cerebellum, and retina DHSs. Distribution of DHSs present in mature cerebral cortex and cerebellum brain regions and mature retina relative to genomic features. (B) Gene Ontology Molecular Function enrichment of CNS-core DHSs. (C) Gene Ontology analysis from GREAT, Molecular Function category, of the CNS-core set of DHSs showing enrichment near neuronal genes. (JPEG 262 KB) [file 13072_2014_358_MOESM2_ESM.jpeg]

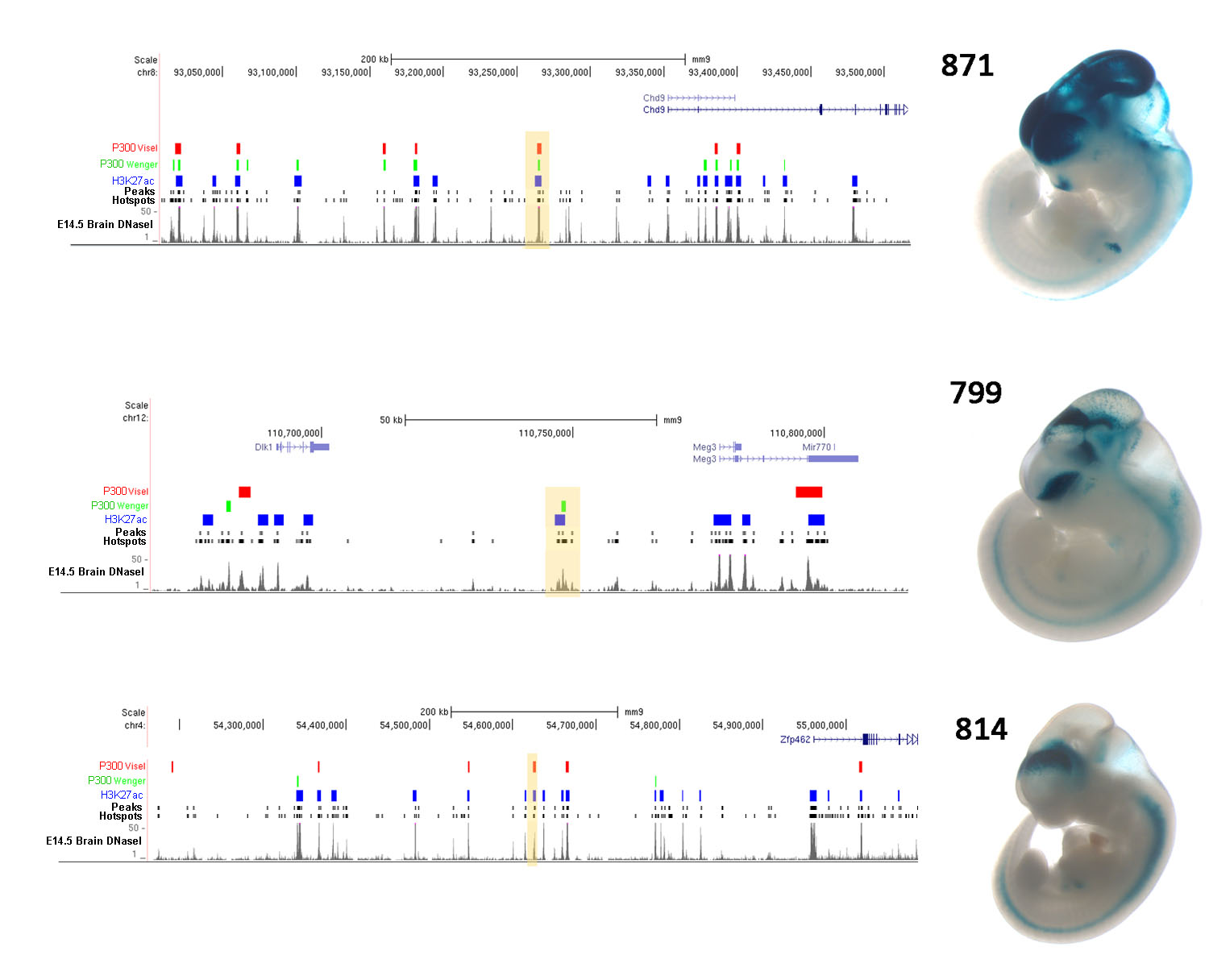

Supplement: Supplementary file 4 — Additional file 4: Figure S4: DNase I hypersensitivity corresponds to enhancer regions identified in previous studies and confirmed in transgenic mice for the VISTA Browser project [30]. Three different enhancers with their expression patterns are shown at the right in transgenic mice and as a tan-shaded region in the UCSC browser tracks. The P300 and H3K27ac ChIP-seq peaks from previous studies are labeled as in Figure 1D. The mm871 enhancer (tan shaded) shows overlap with the DNase I peak, the P300 ChIP-seq peaks, and the H3K27ac peak, whereas the other two enhancers show a DNase I hotspot and two of the three other marks. (JPEG 148 KB) [file 13072_2014_358_MOESM4_ESM.jpeg]

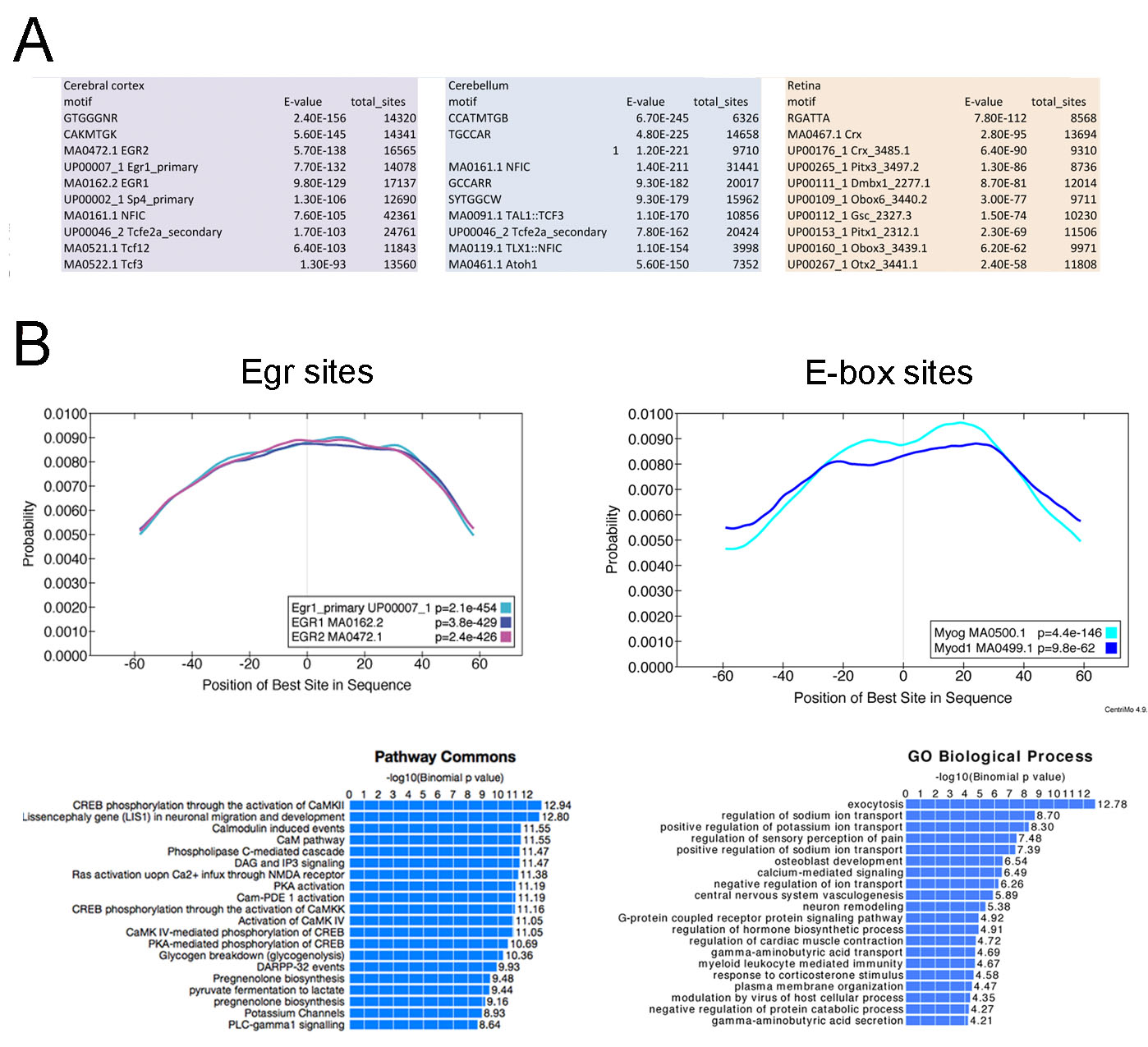

Supplement: Supplementary file 5 — Additional file 5: Figure S5: Sets of enriched DHSs between CNS tissues and CentriMo analysis. (A) The sets of DHSs enriched for the cerebral cortex, cerebellum, or retina were analyzed with the MEME suite (DREME and CentriMo), and we found a distinct pattern of enrichment for transcription factor motifs in the different sets. EGR1 sites and bHLH transcription factor sites were highly enriched in the cerebral cortex, whereas Crx sites were over-represented in DHSs from the retina. (B) CentriMo analysis for EGR1 and E-box sites in cerebral cortical DHSs shows enrichment near the central regions of these DHSs for the over-represented transcription factor motifs, consistent with their role as enhancers. Below: GO enrichment terms associated with EGR1 and E-box sites in the cortex. (JPEG 284 KB) [file 13072_2014_358_MOESM5_ESM.jpeg]

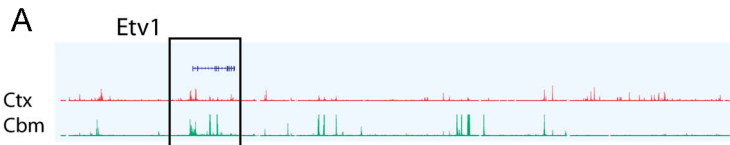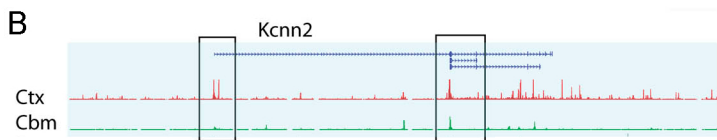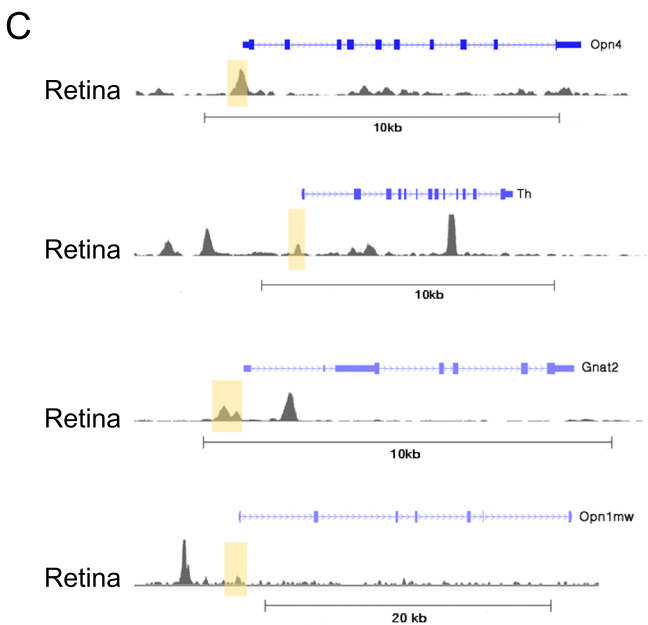

Supplement: Supplementary file 6 — Additional file 6: Figure S6: DNase I hypersensitivity at the promoters of cell type-specific genes. DNase I landscape (cerebral cortex, Ctx (red); cerebellum, Cbm (green)) surrounding the gene bodies of (A) Etv1 and (B) Kcnn2 and accompanying in situ data (A’-A”’, B’-B”). Black arrows point to cell layers with positive (dark purple) in situ signal. In situ data from 2014 Allen Institute for Brain Science. Available from http://mouse.brain-map.org/[66]. (C) DNase I landscape from the P0 retina for cell type-specific genes: Opn4 (ganglion cells), Th (amacrine cells), Gnat2 and Opn1mw (cone photoreceptors). Tan box indicates DHS at the promoter of each gene. (PDF 4 MB) [file 13072_2014_358_MOESM6_ESM.pdf]

A

## Retinal Specific DHSs - GO Biological Process

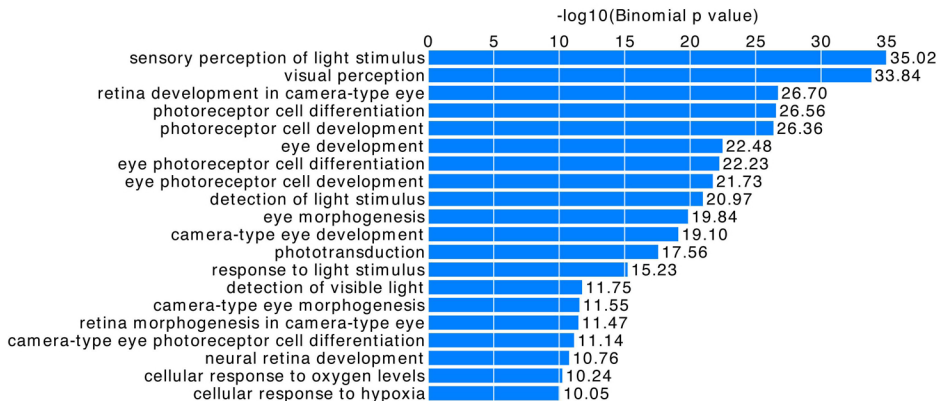

B

## Retinal Specific DHSs - Disease Ontology

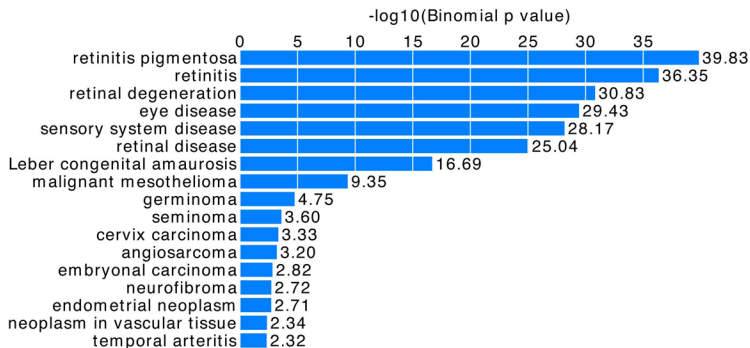

Supplement: Supplementary file 8 — Additional file 8: Figure S8: Gene ontology enrichment of retinal specific DHSs. Gene ontology (A) biological process and (B) disease ontology categories for genes associated with retina-specific DHSs as determined by GREAT analysis. (PDF 3 MB) [file 13072_2014_358_MOESM8_ESM.pdf]

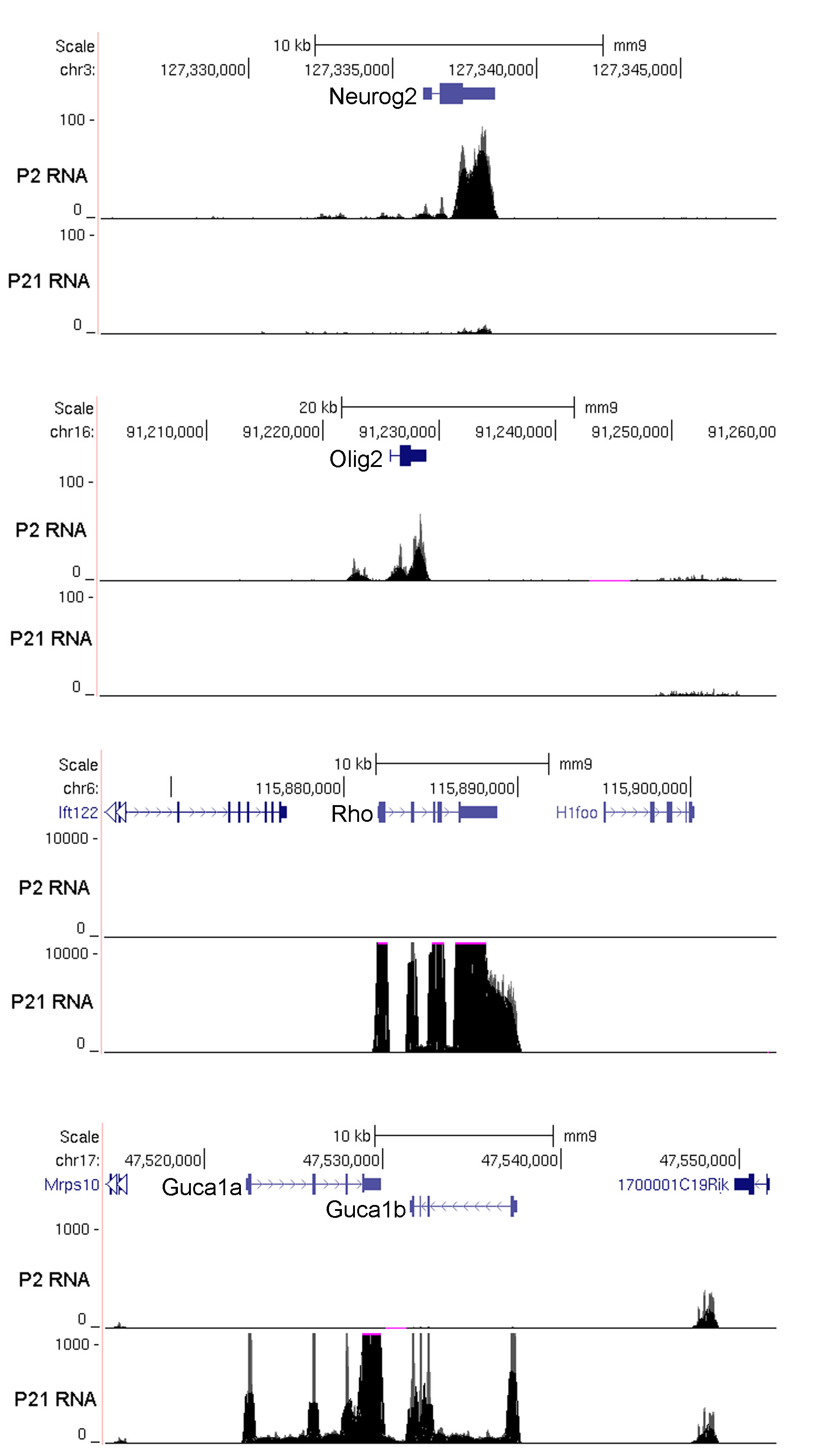

Supplement: Supplementary file 9 — Additional file 9: Figure S9: (Related to Figure 4A,B) RNA-seq landscape for P2 and P21 retina surrounding two progenitor genes expressed in the early retina: Neurog2 and Olig2, and three photoreceptor genes expressed in the mature retina: Rho and Guca1a/b. RNA-seq data previously generated by [45]. (JPEG 213 KB) [file 13072_2014_358_MOESM9_ESM.jpeg]

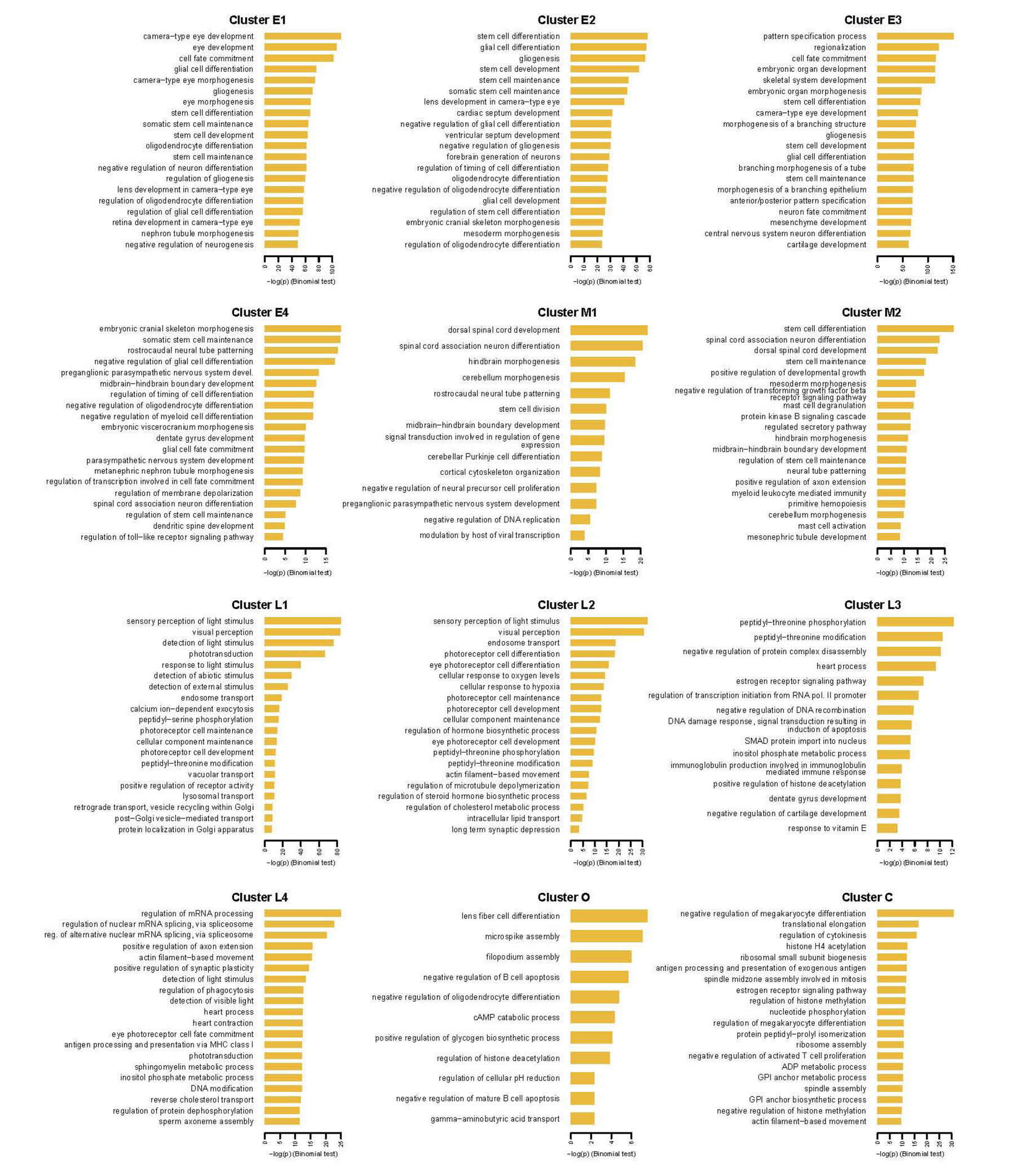

Supplement: Supplementary file 10 — Additional file 10: Figure S10: Gene ontology analysis of genes near k-means-clustered DHSs from retina tissue. Gene ontology (biological process) categories for genes associated with DHSs within each k-means temporal cluster of retinal DHSs (P0, P7, and adult retina) as determined by GREAT analysis. E, early clusters; M, mid-clusters; L, late clusters; O, other cluster groups; C, constitutive cluster group. (JPEG 579 KB) [file 13072_2014_358_MOESM10_ESM.jpeg]

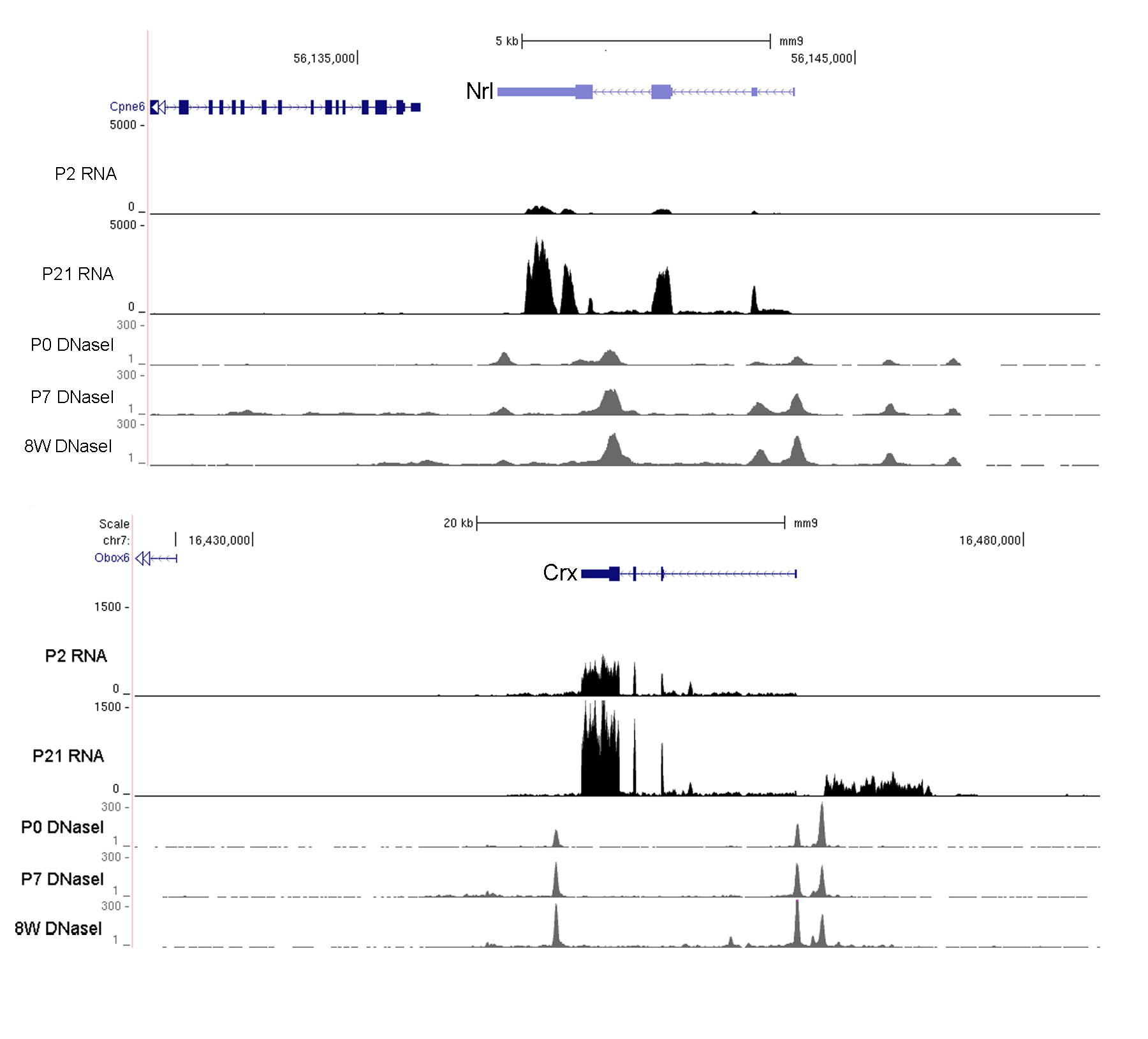

Supplement: Supplementary file 11 — Additional file 11: Figure S11: (Related to Figure 4D,E) The RNA-seq landscape for P2 and P21 retina and the DNase I landscape for P0, P7, and 8-week adult (8w) retina surrounding two retinal development and differentiation genes: Nrl and Crx. RNA-seq data previously generated by [45]. (JPEG 137 KB) [file 13072_2014_358_MOESM11_ESM.jpeg]

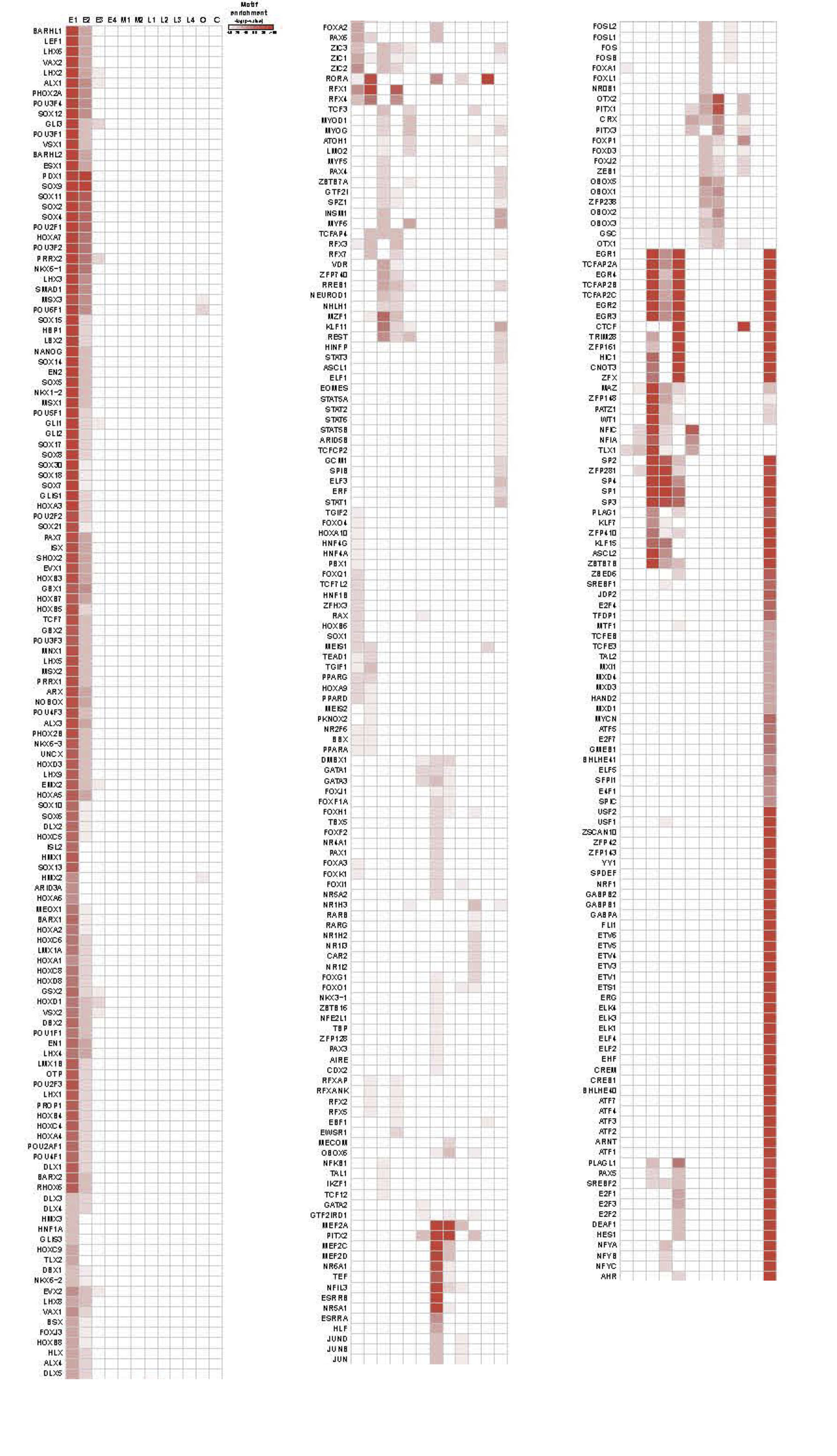

Supplement: Supplementary file 12 — Additional file 12: Figure S12: Transcription factor binding motif enrichment within temporal clusters of total retinal DHSs. Transcription factor binding motif enrichment (-log(P value)) indicated as color intensity for each transcription factor (rows) within each temporal cluster group (columns) from P0, P7, and adult stages of mouse retina. E, early clusters; M, mid-clusters; L, late clusters; O, other cluster groups; C, constitutive cluster group. (JPEG 694 KB) [file 13072_2014_358_MOESM12_ESM.jpeg]

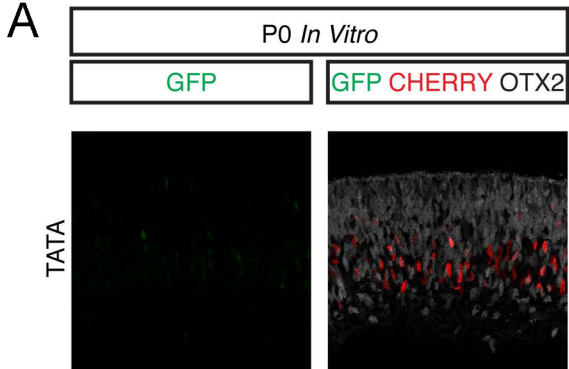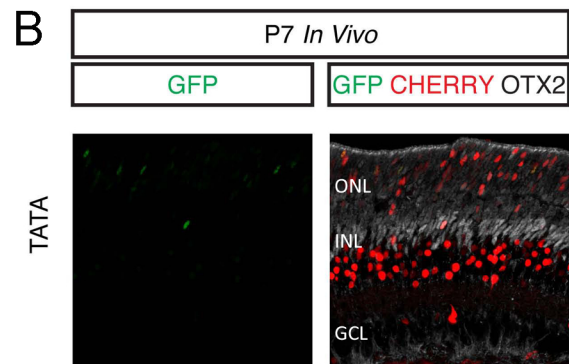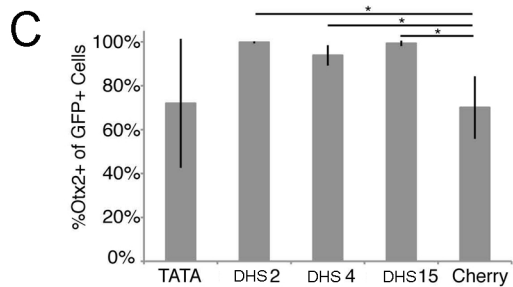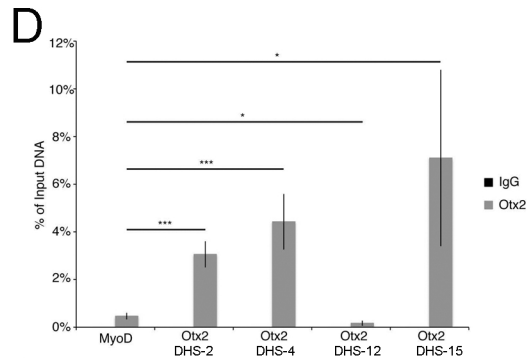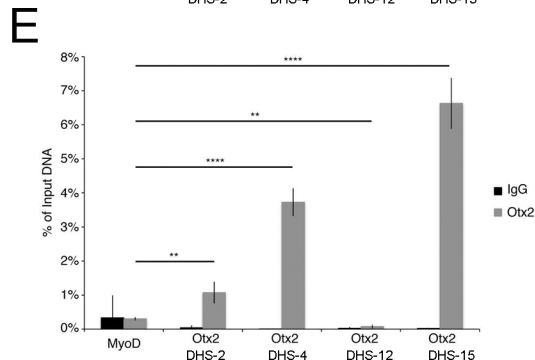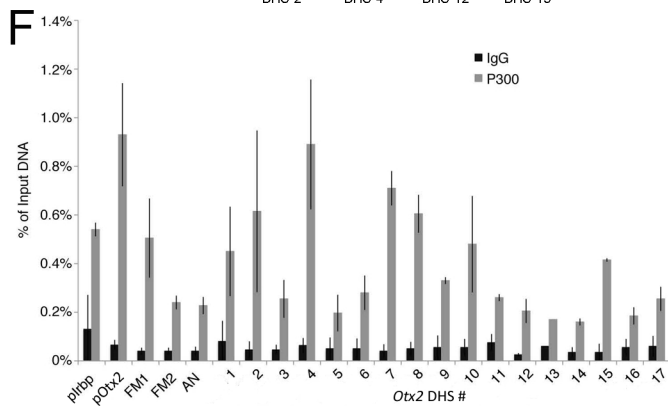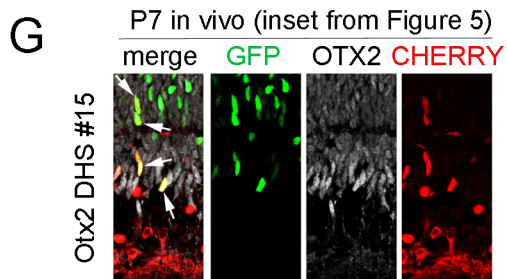

Supplement: Supplementary file 14 — Additional file 14: Figure S14.: Otx2 DHS reporter expression and transcription factor binding. (A, B) Panels show representative images of expression from empty minimal reporter constructs (TATA) with no Otx2 DHS insert (green) co-immunostained for transfection control plasmid (CHERRY, red) and endogenous OTX2 (white). (A) Expression from TATA in electroporated P0 retinal explants cultured 24 h in vitro (N = 3). (B) Expression from TATA in retinas electroporated in vivo at P0 and harvested at P7 (N = 3). ONL, outer nuclear layer; NBL, neuroblastic layer; GCL, ganglion cell layer; INL, inner nuclear layer. (C) Quantification of the percentage of GFP+ cells that co-express OTX2+ for Otx2 DHSs, non-specific control plasmid (TATA), and transfection control (CHERRY) in P7 retina in vivo (N = 2 to 5). *P < 0.01; error bars ± SD. (D, E) Chromatin immunoprecipitation for OTX2 or IgG control from P0 (D) and Adult (E) whole retina tissue shown as a percentage of input DNA. Assayed regions are Otx2 DHSs 2, 4, 12, and 15 with the MyoD promoter serving as a negative control. N = 3 ± S.D. *P < 0.05; **P < 0.01; ***P < 0.001, ****P < 0.0001. Error bars ± SD. (F) Chromatin immunoprecipitation for P300 or IgG control shown as a percentage of input DNA for Otx2 DHSs, the Otx2 promoter (pOtx2), previously described enhancers (FM1, FM2, AN), and a positive control promoter (pIrbp). N = 2 to 4 ± S.D. (G) Inset from Figure 5G showing separated color channels and the colocalization of expression from Otx2 DHS #15 reporter construct (green) co-immunostained for transfection control plasmid (CHERRY, red) and endogenous OTX2 (white). Arrows indicate examples of triple positive labeled cells. (PDF 3 MB) [file 13072_2014_358_MOESM14_ESM.pdf]
