# Supplementary material for: DNase I hypersensitivity analysis of the mouse brain and retina identifies region-specific regulatory elements
Source: Epigenetics Chromatin. 2015 Feb 28;8:8. doi: 10.1186/1756-8935-8-8 (PMC4429822; doi:10.1186/1756-8935-8-8)
Supplement: Supplementary file 13 — Additional file 13: Table S4: Retina-specific DHSs: transcription factor binding motif enrichment. Transcription factor binding motif enrichment from DHSs unique to the mouse retina (not present in other mouse tissues). (DOCX 64 KB) [file 13072_2014_358_MOESM13_ESM.docx]

Supplemental Table S4: Retina Specific DHSs: Transcription Factor Binding Motif Enrichment.

| **TF** | **p-value .adj** | **invLogP** |
| --- | --- | --- |
| OTX2 | 6.98E-41 | 92.5 |
| PITX3 | 1.03E-30 | 69.0 |
| CRX | 1.65E-30 | 68.6 |
| PITX1 | 3.36E-29 | 65.6 |
| OBOX1 | 4.24E-23 | 51.5 |
| DMBX1 | 2.73E-20 | 45.0 |
| GTF2IRD1 | 1.45E-19 | 43.4 |
| OBOX3 | 1.76E-19 | 43.2 |
| OBOX6 | 1.56E-18 | 41.0 |
| PITX2 | 5.75E-18 | 39.7 |
| OBOX5 | 2.08E-15 | 33.8 |
| OBOX2 | 5.91E-14 | 30.5 |
| RORA | 1.14E-12 | 27.5 |
| RAX | 1.24E-12 | 27.4 |
| GSC | 3.26E-11 | 24.1 |
| OTX1 | 2.69E-10 | 22.0 |
| GATA1 | 1.67E-09 | 20.2 |
| MEF2A | 1.42E-08 | 18.1 |
| GSX2 | 2.93E-05 | 10.4 |
| GATA3 | 1.94E-04 | 8.5 |
| NR1H4 | 1.77E-03 | 6.3 |
| NKX2-3 | 5.05E-03 | 5.3 |
