# Supplementary material for: DNase I hypersensitivity analysis of the mouse brain and retina identifies region-specific regulatory elements
Source: Epigenetics Chromatin. 2015 Feb 28;8:8. doi: 10.1186/1756-8935-8-8 (PMC4429822; doi:10.1186/1756-8935-8-8)
Supplement: Supplementary file 15 — Additional file 15: Table S5: P values resulting from pairwise chi-squared on DHS distribution into conservation categories. Related to Figure 5J. (DOCX 43 KB) [file 13072_2014_358_MOESM15_ESM.docx]

Supplemental Table S5: P-values resulting from pair-wise Chi-Squared on DHS distribution into conservation categories. Related to Figure 5J.

|  | Cerebellum | CNS | CNS-core | Cortex | Brain |
| --- | --- | --- | --- | --- | --- |
| CNS | 0 |  |  |  |  |
| CNS-core | 1.33E-51 | 6.45E-138 |  |  |  |
| Cortex | 0 | 0 | 2.44E-43 |  |  |
| Brain | 4.18E-183 | 0 | 3.13E-43 | 5.16E-82 |  |
| Retina | 1.99E-34 | 3.26E-226 | 5.82E-73 | 0 | 0 |
