# Supplementary material for: DNase I hypersensitivity analysis of the mouse brain and retina identifies region-specific regulatory elements
Source: Epigenetics Chromatin. 2015 Feb 28;8:8. doi: 10.1186/1756-8935-8-8 (PMC4429822; doi:10.1186/1756-8935-8-8)
Supplement: Supplementary file 16 — Additional file 16: Table S6: Mouse DHS alignment and conservation with human DHSs. The numbers of mouse DHSs falling into each category of alignment and DHS conservation in humans. (DOCX 77 KB) [file 13072_2014_358_MOESM16_ESM.docx]

Supplemental Table S6: The numbers of mouse DHSs falling into each category of alignment and DHS conservation in human.

| Tissue | Conservation Category | # Of DHSs |
| --- | --- | --- |
| CNS | Not Aligned in Human | 19799 |
| CNS | Aligned But Not DHS in Human | 8140 |
| CNS | Aligned And DHS in Human | 45920 |
| Retina | Not Aligned in Human | 43739 |
| Retina | Aligned But Not DHS in Human | 22460 |
| Retina | Aligned And DHS in Human | 82140 |
| CNS-core | Not Aligned in Human | 853 |
| CNS-core | Aligned But Not DHS in Human | 1011 |
| CNS-core | Aligned And DHS in Human | 2470 |
| Cerebellum | Not Aligned in Human | 30718 |
| Cerebellum | Aligned But Not DHS in Human | 17871 |
| Cerebellum | Aligned And DHS in Human | 57246 |
| Cerebrum | Not Aligned in Human | 64761 |
| Cerebrum | Aligned But Not DHS in Human | 51772 |
| Cerebrum | Aligned And DHS in Human | 107182 |
| Whole Brain | Not Aligned in Human | 66172 |
| Whole Brain | Aligned But Not DHS in Human | 46597 |
| Whole Brain | Aligned And DHS in Human | 111432 |
